# Supplementary material for: Gene editing therapy as a therapeutic approach for cardiovascular diseases in animal models: A scoping review
Source: PLoS One. 2025 Jun 4;20(6):e0325330. doi: 10.1371/journal.pone.0325330 (PMC12136301; doi:10.1371/journal.pone.0325330)
Supplement: S1 File — (DOCX) [file pone.0325330.s001.docx]

**Identification of studies via databases and registers**

Record identified from:

- PubMed: 782

- Science Direct: 145

- Web of Science: 68

Total: n = 995

Records removed *before screening*:

- Duplicate records removed (n = 152)

**Identification**

Records excluded (n = 673)

- Book chapter: n = 82

- Review: n = 188

- Letter to Editor: n = 27

- Poster/abstract: n = 43

- Communiaction/brief report: n = 25

- Not relate to cardiovascular disease (n= 304)

Title and abstracts screened.

(n = 843)

**Screening**

Reports excluded (n = 120):

- lack of clear description of gene editing therapy (n = 34)

- Gene knock-in/knock-out to make disease models without clearly presenting the treatment effects (n = 86)

Reports assessed for eligibility

(n = 170)

Studies included in review

(n = 50)

**Included**
