# Supplementary material for: Gene editing therapy as a therapeutic approach for cardiovascular diseases in animal models: A scoping review
Source: PLoS One. 2025 Jun 4;20(6):e0325330. doi: 10.1371/journal.pone.0325330 (PMC12136301; doi:10.1371/journal.pone.0325330)
Supplement: S2 File — (DOCX) [file pone.0325330.s002.docx]

**Gene editing therapy as a therapeutic approach for cardiovascular diseases in animal models: a scoping review**

**Searching keys:**

("gene editing" OR "CRISPR" OR "Prime editing" OR "Base editing" OR "TALEN" OR "ZFN" OR "genome editing") AND ("cardiovascular disease" OR "heart failure" OR "cardiomyopathy" OR "myocardial infarction" OR "coronary artery disease" OR "hypercholesterolemia" OR "atherosclerosis" OR "amyloidosis" OR "duchenne muscular dystrophy") AND ("animal model" OR "mice" OR "mouse" OR "rat" OR "rabbits" OR "pig" OR "swine" OR "primates" OR "in vivo")

Table S1. Detail outcomes of included studies.

| No | Author | Treated | Control | Randomization | Blinding | Species and Strain | Age | Disease Model | Target Gene | Gene Editing Tool | Delivery Method | Dosage and Frequency | Primary Outcomes: | |
| --- | --- | --- | --- | --- | --- | --- | --- | --- | --- | --- | --- | --- | --- | --- |
| 1 | Alba Carreras, 2019 (1) | n= 8 per group | n =6–8 per group | Yes | Yes | mice | 10 - 28 weeks | Hypercholesterolemia | PCSK9 | CRISPR-Cas9 and BE3 (cytidine base editor) | AdV | Tail vein injection, 1×10⁹ viral particles | Primary Outcomes: Editing Efficiency: Cas9 and BE3 induced efficient editing at respective loci BE3: high rate of C-to-T transitions at W159 codon Cas9: induced mostly frameshift/in-frame INDELs Editing % (human PCSK9): 10–34% (Cas9), 11–35% (BE3) BE3 produced more precise nonsense mutations with fewer variants Lipid Levels: Significant ↓ in total and LDL cholesterol with: Cas9-gH BE3-gMH Cas9-gM (mouse gene) alone had no significant effect on cholesterol in hPCSK9-KI mice Protein Expression: ↓ human PCSK9 protein levels (ELISA) after both Cas9 and BE3 editing ↓ LDL-cholesterol mirrored ↓ PCSK9 levels Histology: Liver staining: ↓ PCSK9 protein, ↑ LDL receptor (LDLR) No abnormal morphology, inflammation, or damage reported Off-target Effects: GUIDE-seq for Cas9-gH: 2 weak off-targets (intergenic, intronic) CIRCLE-seq + amplicon sequencing: No off-targets detected in BE3-treated mice at 24 predicted sites Translocation: Detected in Cas9-treated mice, but not in BE3-treated) Secondary Outcomes: Adverse Outcomes No toxicity, inflammation, or abnormal pathology No off-target edits or chromosomal translocations from BE3 Cas9-induced chromosomal rearrangements detected between mouse and human loci BE3 showed higher precision and safety profile | |
| 2 | Alexandra C. Chadwick, 2018 (2) | n= 5 - 9 per group | n = 5 | Not mention | Not mention | mice | 5 weeks | Hyperlipidemia | ANGPTL3 | CRISPR-Cas9 base editing (Base Editor 3 - BE3) | Adenoviral vector | Not mention | Primary Outcomes: Editing Efficiency Liver editing at Angptl3 locus: 35% median editing rate at day 7 Deep sequencing of 10 predicted off-target sites: no evidence of editing Plasma Lipid Levels: Triglycerides: Reduction by 31% in BE3-Angptl3-treated mice (compared to control). Reduction by 56% in Ldlr-knockout mice. Cholesterol: Reduction by 19% in BE3-Angptl3-treated mice. Reduction by 51% in Ldlr-knockout mice. Base Editing Efficiency: Median editing rate of 35% at the Angptl3 target site. Secondary Outcomes: Bone Marrow Hematopoietic Stem Cells: No decrease observed compared to control Adverse Outcomes No off-target edits detected at top 10 CRISPOR-predicted sites No signs of hematopoietic toxicity or systemic inflammation No adverse outcomes reported post-injection | |
| 3 | Andreas C. Chai, 2023 (3) | n = 5 per group | n = 5-8 per group | Yes | Yes | mice | P0 (postnatal day 0) | Hypertrophic Cardiomyopathy ( | MYH7 c.1208G>A (p.R403Q) | ABEmax-VRQR | Dual AAV9 vectors | Low dose: 8 × 10¹³ vg/kg High dose: 3 × 10¹⁴ vg/kg Single dose administration | Primary Outcomes: Editing Efficiency: cDNA editing in heart: 35% in homozygous model, 12.9–26.7% across heart chambers in heterozygous mice DNA editing in heart: 5.5–8.0% Off-target editing: DNA: <0.12% in non-cardiac tissues RNA: No increase in transcriptome-wide A-to-I editing No editing in homologous MYH6 gene Survival and Function in Homozygous Mice: Saline-treated Myh6^h403/h403: median survival 7 days ABE high-dose treated: median survival extended to 15 days Moderate rescue of early mortality Prevention of Disease in Heterozygous Mice: ABE-treated Myh6^h403/+ had normal: Wall thickness Ventricular dimensions Ejection fraction and fractional shortening Heart weight/tibia length, fibrosis area, histology — similar to WT Untreated Myh6^h403/+ showed HCM phenotype with increased wall thickness, fibrosis, and ventricular remodeling Functional and Molecular Rescue: Normalized contractile force and ATP consumption in iPSC-CMs from HCM patients after correction RNA-seq: ABE-treated mice showed reversal of 257 DEGs associated with disease; expression similar to WT Normalization of hypertrophic gene Nppa (2.8× higher in diseased, normalized after editing) Secondary Outcomes: Adverse Outcomes No increased RNA editing or off-target DNA mutations No systemic toxicity No functional effects in WT or corrected mice Protein expression of sarcomeric proteins unaffected | |
| 4 | Bin Li, 2021 (4) | n = 5 | n = 5 | Not mention | Yes | mice | postnatal day 3; | Dilated Cardiomyopathy | XIRP1 | Gene overexpression | AAV9 vector | 1 × 10¹² vg per mouse 1 × 10¹² vg per mouse Intraperitoneal injection 1 × 10¹² vg per mouse | Primary Outcomes:  Evaluated Outcomes Molecular and Histological Improvements: ↑ XINB expression in TNNT2-ΔK210 cardiomyocytes ↓ myofilament disorganization ↓ ventricular dilation and interstitial fibrosis (Masson's staining) Improved ultrastructural organization (Z-line alignment, mitochondria, sarcomeres) Functional Rescue: Echocardiography at 3 months: ↑ Ejection Fraction from 64.3 ± 3.7% to 78.6 ± 3.1% ↑ Fractional Shortening from 28.8 ± 3.1% to 40.0 ± 2.8% ↓ LV end-systolic diameter from 2.5 ± 0.2 mm to 2.0 ± 0.06 mm ↓ LV end-diastolic diameter from 4.3 ± 0.2 mm to 3.8 ± 0.1 mm Cardiomyocyte Contractility (in vitro): XINB overexpression enhanced spontaneous contractile force ↑ expression of TNNT2, ACTN2, and MYH7 ↓ disorganized cTnT pattern ↑ multinucleation (suggesting maturity) Secondary Outcomes: Adverse Outcomes No adverse events or toxicity observed from AAV9-XINB delivery No fibrosis, inflammation, or off-target gene expression outside heart Bioluminescence confirmed heart-specific transgene expression | |
| 5 | Camilo Breton, 2020 (5) | Mice: n=5 per group  NHPs: n=2–3 per group | n= 3 | Not mention | Yes | mice and NHP | Mice: ~8 weeks NHPs: Adults | Hypercholesterolemia | PCSK9 | M2PCSK9 meganuclease (engineered I-CreI) | AAV8 vector | Mice: 1 × 10¹¹ GC/mouse (after hPCSK9 pre-injection) NHPs: 6 × 10¹² or 3 × 10¹³ GC/kg intravenously | Primary Outcomes: Evaluated Outcomes On-Target Editing: In mice: Parental AAV8.M2PCSK9: up to 67% indel at PCSK9 site (week 9) Modified AAVs (self-targeting, short-promoter): 18–41% indels In NHPs: On-target indels: 15–43% (day 18), decreased by day 128 Most edits were AAV-derived insertions PCSK9 and LDL Levels: Serum PCSK9: AAV8.M2PCSK9: ↓ to 40% of baseline AAV8.Target.M2PCSK9, AAV8.TBG-S1-F113.M2PCSK9: similar or better reductions LDL-C: AAV8.TBG-S1-F113.M2PCSK9: ↓ to 64–74% of baseline Higher dose AAV8.MutTarget.M2PCSK9+PEST: ↓ to 61% Off-Target Editing: Mice (ITR-seq): AAV8.M2PCSK9: avg. 161 off-targets Modified AAVs: ~6–20-fold fewer off-targets NHPs (ITR-seq + AMP-seq): AAV8.M2PCSK9: avg. 131 off-targets at day 18 AAV8.TBG-S1-F113.M2PCSK9: 6-fold fewer off-targets Indel% at off-target sites: generally <1% Immune Response & Toxicity: Mild T-cell response to M2PCSK9 in some NHPs AAV8.M2PCSK9 group: ↑ ALT (up to 1,112 U/L) Modified AAVs: much lower ALT/AST levels No systemic toxicity or significant adverse effects reported Secondary Outcomes: AAV8.M2PCSK9 had higher: ALT/AST elevations Off-target edits T-cell responses Modified AAVs (self-targeting, PEST, short promoter) minimized these effects | |
| 6 | Chengzu Long, 2016 (6) | n = 12 | n = 3–4 per group | Not mention | Yes | mice | postnatal day 1, day 12 or day 18 | Duchenne Muscular Dystrophy | DMD | CRISPR-Cas9 (SpCas9) | AAV9 vectors: | Intramuscular (IM) at P12 Retro-orbital (RO) at P18 Intraperitoneal (IP) at P1 | Primary Outcomes: Evaluated Outcomes Editing and Exon Skipping: CRISPR-Cas9 targeted exon 23 using non-homologous end joining (NHEJ) Efficient exon 23 deletion (ΔEx23) confirmed by RT-PCR and sequencing Restored splicing from exon 22 to exon 24 Dystrophin Expression (at 6–12 weeks post-treatment): Immunohistochemistry (% of dystrophin-positive fibers): IM: ~25.5% (TA muscle) RO: ~6.1% (TA), ~9.6% (heart) IP: ~1.8% (TA), ~3.2% (heart) Protein levels (semiquantitative IHC, % of WT): IM: ~53% (TA), ~70% (heart) RO: ~28% (TA), ~71% (heart) IP: ~24% (TA), ~52% (heart) Functional Recovery: Grip Strength (4 weeks post IP-AAV injection): mdx untreated: ~35% of WT mdx + AAV-CRISPR: ~48% of WT WT: ~72% Histology: ↓ necrotic myofibers ↓ inflammatory infiltration ↑ fiber integrity in treated mice vs untreated Off-Target Analysis: T7E1 assay of top 10 predicted off-targets: No cleavage detected in any sites except on-target site No evidence of editing in germline (sperm analysis) Secondary Outcomes: No overt toxicity or adverse pathology No detectable germline transmission No off-target editing among top-predicted sites Did not restore dystrophin in CNS due to blood-brain barrier | |
| 7 | Daniel Reichart, 2023 (7) | n = 3 - 6 per group | n =4 - 12 per group | Yes | Yes | mice | postnatal day 10–13 | Hypertrophic Cardiomyopathy | MYH6 R403Q | CRISPR-Cas9 (SaCas9) and ABE8e | Dual AAV9 vectors | ABE8e: 1.25 × 10¹³ vg/kg per AAV (single or double dose) Cas9: Low dose: 1.1 × 10¹² vg/kg Medium: 5.4 × 10¹² vg/kg  High: 1.1–2.2 × 10¹³ vg/kg | Primary Outcomes: Editing Efficiency: ABE8e: cDNA (LV): 81% ± 8% (single dose), 68% ± 6% (two doses) gDNA (LV): ~16% Atrial editing improved with two doses (LA: from 26% to 50%) Cas9: Inactivation in LV: up to 72% (dose-dependent) WT allele off-target disruption: ~9% at high dose Cardiac Functional Outcomes: ABE8e: Prevented LV hypertrophy, fibrosis, and contractile dysfunction Restored normal echocardiographic indices (LV wall thickness, FS%) Reversed transcriptional changes linked to hypertrophy and fibrosis Cas9: High dose prevented hypertrophy but caused contractile depression Medium dose optimal (balanced inactivation and safety) Low dose inconsistent Histology and Transcriptomics: ABE8e: Masson's trichrome: significantly reduced fibrosis RNA-seq: normalized metabolic, contractile, and fibrotic gene expression Cas9: Histology normal with medium/low dose High dose caused contractility loss in ~30% of cardiomyocytes Secondary Outcomes: Adverse Outcomes: ABE8e: Mild bystander edits at A10, A11 (~5–10%) Minimal off-target DNA editing (only 3/16 sites <0.4%) No RNA off-target editing Cas9: High dose: WT allele disruption, impaired function, 2 mice developed heart failure Medium dose: safe and effective No editing observed in non-cardiac tissues (liver, lung, etc.) | |
| 8 | F Ann Ran, 2015 (8) | n = 3 per group | n = 3–5 per group | Yes | Yes | mice | 5–6 weeks | Hypercholesterolemia | PCSK9 | CRISPR-Cas9 (SaCas9) | AAV8 vector | Single intravenous injection 0.5 × 10¹¹ to 4 × 10¹¹ vg per animal | Primary Outcomes: Editing Efficiency: >40% indel formation in liver tissue one week post-injection Indels maintained at 2 and 4 weeks Histological lipid accumulation seen in Apob-targeted mice (as validation) Biochemical Impact: Serum Pcsk9: ↓ ~95% at all doses Total cholesterol: ↓ ~40% Effects sustained over 4 weeks Specificity & Off-target Analysis: BLESS used for genome-wide DSB detection Very low DSB signals outside the on-target site Targeted sequencing of top BLESS-predicted off-targets: no detectable indels in vivo No indels at off-targets predicted by sequence similarity either Toxicity & Immune Response: No abnormalities on liver necropsy No inflammation on H&E staining No elevated ALT, AST, albumin, or bilirubin Slight increase in AST at 4 weeks, but within normal range and seen across all groups Secondary Outcomes: No hepatic toxicity No detectable off-target editing No immune reaction or systemic illness within 4-week period | |
| 9 | Handan Hu, 2022 (9) | n = 4 | n = 4 | Not mention | Not mention | rats | postnatal 7 days | familial atrial fibrillation | MYL4 E11K/E11K | genetic expression | AAV9 | 1×10¹² total vector particles (tvp)/kg | Primary outcomes Myl4 Protein Expression: Western blot showed restoration of Myl4 protein levels in treated Myl4 E11K/E11K rats to levels comparable to wild-type rats. ECG Analysis: AAV9-cTNT-Myl4 rescued the P-waves and PR intervals. PR interval at 6 months: Significantly reduced compared to untreated controls. Regular RR intervals and P-wave restoration sustained up to 12 months post-treatment. Transthoracic Echocardiography: Left atrium diastolic diameter: Treated group: Significantly smaller (4.03 mm at 3 months; 5.48 mm at 12 months) compared to untreated rats. Left ventricular ejection fraction (LVEF): Comparable to wild-type rats at 3 months and improved at 12 months compared to untreated controls. Secondary Outcomes: Inflammation and Fibrosis: Significant reduction in pro-inflammatory cytokines (TNF-α, IL-1β, TGF-β1) levels. Masson's trichrome staining showed reduced atrial fibrosis in treated rats. Immunostaining of α-SMA confirmed reduced fibrosis levels. Myocyte Disarray: H&E staining revealed improved atrial cardiomyocyte alignment in treated rats. Connexin 43 (Cx43) Localization: Restored Cx43 localization at intercalated discs in atrial tissues. Adverse Outcomes No evidence of: Ventricular toxicity Inflammation in ventricular tissue Adverse histological changes Long-term expression of Myl4 observed up to 12 months No adverse events reported with AAV9-Myl4 injection | |
| 10 | Hao Yin, 2017 (10) | n = 4–6 per group | n=5 per groups | Not mention | Yes | mice | 8–10 weeks | Hereditary tyrosinemia type I | PCSK9  FSH and ROSA26 | CRISPR-Cas9 | Lipid nanoparticle | Cas9 mRNA: 1.2 mg/kg sgRNA: 0.5 mg/kg (for GFP/Pcsk9) or 0.4 mg/kg (for Fah) Single intravenous injection, with an optional second dose after 5 days in some experiments | Primary Outcomes: Evaluated Outcomes Editing Efficiency (by TIDE, T7EI, and deep sequencing): Pcsk9: e-sgRNA (2 guides): ~83% editing, including small indels, large deletions Unmodified sgRNA: ~5% 5′&3′ sgRNA: ~25% Fah: e-sgRNA: >40% editing Unmodified and 5′&3′ sgRNA: ~14% AAV-delivered sgRNA: ~24% ROSA26: e-sgRNA: significantly higher than other groups (~35–50%) Biochemical Impact: Serum Pcsk9: undetectable 5 days post injection Serum total cholesterol: ↓ 35–40% Histology & Organ Specificity: Editing detected only in hepatocytes, not in non-parenchymal liver cells or other organs (lung/spleen) No inflammation or histologic damage in liver Liver enzyme levels (ALT, AST, bilirubin) remained normal Immune Response: IL-6 and G-CSF induced by native and 5′&3′ sgRNA but not by e-sgRNA Suggests lower innate immune activation with chemical modification Off-target Effects (GUIDE-seq and deep sequencing): Very low off-target activity For Pcsk9-2, only one site had 1% off-target indels with e-sgRNA e-sgRNA had improved specificity ratio vs plasmid-expressed sgRNA Secondary Outcomes: No detectable toxicity, weight loss, or organ damage No detectable gene editing in lung or spleen Minimal off-target activity with chemically optimized sgRNAs | |
| 11 | Hengzhi Du, 2024 (11) | n=7-8 per group | n=8–10 per group | Not mention | Not mention | mice | 8 weeks | Dilated Cardiomyopathy | CRT (Dilated Cardiomyopathy Repressive Transcript) | CRISPR-Cas9 | AAV9 | Single intravascular or subcutaneous injection | Primary Outcomes: DCRT knockout (DCRT−/−): Induced spontaneous DCM phenotype: ↓ Ejection fraction and fractional shortening ↑ LV dilation, ↑ ANP/BNP, ↑ ROS, ↑ fibrosis ↓ mitochondrial respiration, ↓ ATP, ↑ cardiomyocyte death DCRT overexpression (DCRT-TG or rAAV9-DCRT): Protected against TAC-induced DCM: Improved LV function and reduced dilation Normalized ANP/BNP levels ↓ ROS and fibrosis Restored mitochondrial function Mechanism—DCRT regulates NDUFS2 splicing: Binds PTBP1 in the nucleus to suppress exon 3 skipping of NDUFS2 Loss of DCRT → ↑ NDUFS2-S (short isoform) → ↓ Complex I activity, ↑ ROS, ↓ ATP Overexpression of DCRT or CoQ10 reversed these mitochondrial defects NDUFS2-S overexpression alone: Recapitulated DCM-like phenotypes in WT mice Induced dysfunction in iPSC-CMs and primary cardiomyocytes). Secondary Outcomes: Adverse Effects: No stated off-target effects or systemic toxicity from gene editing or AAV delivery CoQ10 improved function but didn’t restore all antioxidant activity (PRDX5 remained suppressed) | |
| 12 | Huan Zhao, 2020 (12) | n = 6 | n = 6 | Not mention | Not mention | mice | Neonatal mice | Hyperlipidemia | LDLR | CRISPR-Cas9 | AAV8 | AAV-Cas9: 5×10¹⁰ genome copies AAV-sgRNA: 5×10¹¹ genome copies | Primary Outcomes: Gene Editing Efficiency: Homology-directed repair (HDR) achieved in 6.7% of hepatocyte alleles. Indels observed in 25% of Ldlr alleles. LDLR Protein Expression: 18% of wild-type LDLR protein levels restored. 20% of hepatocytes expressed LDLR (immunostaining). Plasma Lipid Levels: Total Cholesterol: Decreased from 40.74 mmol/L to 12.84 mmol/L. Triglycerides: Reduced from 9.12 mmol/L to 4.05 mmol/L. LDL Cholesterol: Reduced from 30.08 mmol/L to 9.98 mmol/L. Atherosclerosis Severity: Atherosclerotic plaque area reduced to 2.78% in treated mice (from ~7% in controls). Plaque perimeter affected: Reduced to 25% (vs. ~87% in controls). Secondary Outcomes: Liver Lipid Accumulation: Oil Red O staining: Hepatic lipid droplets reduced to 7.8% from ~31% in controls. Macrophage Infiltration: Significant reduction in F4/80+ macrophages in atherosclerotic lesions. Histology: Improved smooth muscle cell integrity and reduced fibrosis (Sirius Red staining). | |
| 13 | Jaydev Dave, 2022 (13) | n = 8 | n = 6 | Not mention | Not mention | mice | 2 months | Cardiomyopathy and arrhythmia | PLN-R14del mutation | CRISPR-Cas9 (SaCas9) | AAV9 | 2×10¹² vg AAV9-CRISPR-Cas9-gRNA | Primary out comes Ventricular Geometry and Function: Left Ventricular End-Diastolic Volume (LVEDV): Mutant (hPLN-R14del): 62.7 ± 9.1 µL CRISPR-treated group: 45.4 ± 4.2 µL (P<0.05 vs. mutant) Stroke Volume (SV): Mutant: 49 ± 12.5 µL CRISPR-treated group: 33.6 ± 3.4 µL (P<0.05 vs. mutant) Left Ventricular Ejection Fraction (LVEF): Maintained across groups (~74–81%). Arrhythmia Susceptibility: Ventricular Tachycardia (VT) Threshold: Mutant: 20.3 ± 1.2 Hz CRISPR-treated: 30.9 ± 2.3 Hz (P<0.01 vs. mutant) Control (WT): 25.7 ± 1.3 Hz CRISPR treatment significantly reduced arrhythmia susceptibility under adrenergic stress and pacing. secondary outcomes Adverse Outcomes No toxicity observed in liver or kidney No off-target editing among 5000 screened genomic sites (only 1 intronic variant found, non-coding) No evidence of immune response or Cas9 overexpression outside cardiomyocytes | |
| 14 | Jessie R. Davis, 2022 (14) | n = 3–5 per group | n = 3–5 | Not mention | Yes | mice | 6-8 weeks | Hypercholesterolemia | PCSK9 and Angptl3 | Adenine Base Editors (ABE8e variants) | AAV8 and AAV9 | 1 × 10¹¹ vg/mouse (5 × 10¹² vg/kg), Single injection | Primary Outcomes: Base Editing Efficiency (by HTS): Single-AAV editing of liver (bulk): PCSK9 (humanized mice): 44% Mouse Pcsk9: SaKKH-ABE8e: 54% SaKKH-ABE8e V106W: 47% SauriABE8e: 46% Mouse Angptl3: 61% Heart and Skeletal Muscle (Single-AAV at 8 × 10¹¹ vg): Heart: 33% editing Muscle: 22% editing Functional Effects: Protein Knockdown: PCSK9: 99% knockdown Pcsk9: 91% Angptl3: 94% Plasma Lipid Reduction: Cholesterol: ↓ 24–38% across PCSK9 and Angptl3-targeted groups Triglycerides (Angptl3-edited mice): ↓ 45% Comparisons with Dual-AAV Systems: Single-AAV outperformed dual-AAV especially at low doses and in non-hepatic tissues Editing in heart and muscle was 2.1× and 2.5× higher, respectively, with single-AAV compared to dual-AAV Secondary Outcomes: Histology: No liver toxicity or morphological changes Off-target Editing: Minimal DNA off-targets (≤0.45% at one site) Off-target reduced by TadA V106W mutation No off-target mRNA editing detected Immunogenicity: Not deeply assessed, but no immune toxicity noted AAV Dose Consideration: High AAV doses known to raise concerns in clinical settings | |
| 15 | Jiacheng Li, 2022 (15) | n = 6-8 per group | n = 6 | Yes | Not mention | mice | 6–8 weeks | Cardiac Regeneration Post-Myocardial Infarction and Hypertrophic Cardiomyopathy (HCM) | Meis1 and Hoxb13  MHRT | CRISPR-CasRx | AAV9 | 8×10¹¹ viral genomes | Primary Outcomes: Cardiac Regeneration (Knockdown of Meis1 and Hoxb13): Cardiomyocyte Proliferation: PH3+ (mitosis marker): 4.9-fold increase Ki67+ (cell cycle entry): 4-fold increase Aurora B+ (cytokinesis): 10-fold increase Left Ventricular Function: Improved ejection fraction (EF) post-MI: 48.3% in treated mice vs. 33.1% in controls. Fibrotic scar size reduced by ~60%. Hypertrophic Cardiomyopathy (Knockdown of Mhrt): Cardiac Function: EF decreased to 38% in Mhrt knockdown mice (P<0.01). Increased left ventricular diameter and thinning of ventricular walls. Histology: Fibrosis increased (collagen area: 3-fold higher in treated group). Hypertrophy Indicators: Heart weight-to-body weight ratio increased significantly (P<0.01). Gene Knockdown Efficiency: Meis1: 65.2% knockdown Hoxb13: 83.6% knockdown Mhrt: 61.7% knockdown Secondary outcomes: Adverse Outcomes No morphological abnormalities with CasRx expression alone (assessed in heart, liver, lung, kidney, brain) No apoptosis, DNA damage, or organ toxicity Mhrt knockdown led to functional deterioration (modeling HCM), but not due to vector or CasRx toxicity | |
| 16 | Jing Gong, 2020 (16) | n= 4-6 per group | n= 4-6 per group | Not mention | Not mention | mice | 5 weeks old | Hyperlipidemia | PCSK9  ANGPTL3  APOC3 | CRISPR-Cas9 | lipoMSN | 11 mg/kg ( | Primary Outcomes: Gene Editing Efficiency: Pcsk9: 24.8% indel rate (week 4 post-treatment). Angptl3: 7.2% indel rate. Lipid Profile: Serum Cholesterol: Pcsk9 group: 31.7% reduction. Angptl3 group: 28.2% reduction. Dual-target group (Pcsk9 + Angptl3): 56.5% reduction. Triple-target group: 43.18% reduction. Serum Triglycerides: Angptl3 group: 25% reduction (lasting effect at week 4). LDLR Upregulation: Significant upregulation of LDL receptor expression following Pcsk9 disruption. Secondary Outcomes: Off-Target Effects: Minimal off-target editing observed at predicted loci. Toxicity: No significant changes in weight, HDL-C, or ALT levels. H&E staining showed no observable damage to heart, liver, lung, kidney, or spleen. | |
| 17 | Jonathan D. Finn, 2018 (17) | mice n = 5 per group Rats: n = 5 per group | n = 3–5 | Not mention | Not mention | mice and rats | Mice: 6–10 weeks Rats: 6–8 weeks | Amyloidosis | TTR (transthyretin) | CRISPR-Cas9 (SpyCas9) | LNP-INT01 | Tail vein injectionIIn mice: 0.3, 1.0, or 3.0 mg/kg (single dose) In rats: 1, 2, or 5 mg/kg single dose | Primary Outcomes: Editing Efficiency Mouse liver (CD-1): Up to ~70% editing at genomic DNA level >97% reduction in serum TTR protein Effects lasted ≥12 months Rat liver (Sprague-Dawley): Up to ~70% editing, >90% TTR knockdown Cumulative effect observed with repeat dosing Editing highest in hepatocytes; minor editing in spleen and kidney Functional Effects Serum TTR: Decreased from ~1800 µg/mL to <50 µg/mL Levels stable for 12 months Dose-dependent editing and protein knockdown Editing in pericentral AXIN2+ hepatocyte stem population supports durability Secondary Outcomes: No significant toxicity: No cytokine stimulation No weight loss No immune reactions Lipid LP01 was biodegradable, half-life ~6 hours Cas9 mRNA and sgRNA cleared within 72 hours | |
| 18 | Jonathan M. Levy, 2020 (18) | n = 3–5 per group | n = 3 | Not mention | Yes | mice | 6–9 week | Niemann-Pick Type C disease | NPC1 DNMT1  PCSK9 | Cytosine and Adenine Base Editors (CBE3.9max, ABEmax) | Dual AAV system | Single-dose up to **4 × 10¹² vg per mouse** | Primary Outcomes: Base Editing Efficiency Heart: CBE: 15 ± 3.8% ABE: 20 ± 1.4% Liver: CBE: 21 ± 17% ABE: 38 ± 2.9% Skeletal muscle: CBE: 4.4 ± 2.4% ABE: 9.2 ± 4.0% Brain: Cortex (ABE): up to 87% (sorted cells); ~43% (unsorted) Cerebellum: up to 64% in sorted Purkinje neurons Retina: Photoreceptors: up to 48% (CBE), 37% (ABE) Disease Correction (Npc1 Model) Editing at Npc1 c.3182T>C: High-dose: Cortex: 48% (unsorted), 81% (sorted) Cerebellum: 0.3% (unsorted), 42% (sorted) Phenotypic Rescue: Increased lifespan (median 102.5 to 112 days, p = 0.02) ↑ Purkinje neuron survival: 24% → 38% of WT ↓ CD68+ microglial inflammation Editing specificity: 94% precise C>T edits; minimal indels Only one off-target site detected at 0.3% (intronic, non-coding) Secondary Outcomes: No systemic toxicity reported Long-term expression confirmed Mosaic editing sufficient for partial phenotypic rescue Retinal CBE editing led to some indels (ratio ~1:1), but not overlapping with base-edited alleles | |
| 19 | Kelsey E. Jarrett, 2017 (19) | n = 5 - 6 per group | n = 5 | Yes | Not mention | mice | 6.5–9.5 weeks | Hypercholesterolemia | LDLR and ApoB | CRISPR-Cas9 system (SpyCas9) | AAV8 vector | Intraperitoneal injection of **6 × 10¹¹ GC total per mouse** | Primary Outcomes: Editing Efficiency Liver indel rate: Ldlr: 54.3 ± 16.6% Apob: 74.1 ± 23.4% AAV-ITR integration at the Apob cut site: 10–26% of reads Off-target effects: Ldlr gRNA: ~5% indels at one intronic site (Stx8) Apob gRNA: no detectable off-targets  Phenotypic Effects Plasma Cholesterol: BbsI control: 350 ± 18.7 mg/dL Ldlr-only gRNA: 728 ± 174 mg/dL Ldlr + Apob gRNAs: 125 ± 27.3 mg/dL Atherosclerosis: Lesion area: Ldlr-only: 2.2 ± 2.1% Ldlr + Apob: significantly reduced, nearly absent Control: no lesion Lipoprotein Distribution: Ldlr-only: ↑ VLDL + IDL/LDL cholesterol Ldlr + Apob: ↓ VLDL, IDL/LDL, HDL Liver Lipids: Ldlr + Apob mice had microvesicular steatosis, ↑ liver weight, ↑ triglyceride and cholesteryl ester levels ↑ PLIN2 expression by IHC and Western blot ER Stress Markers: ↑ BIP, GRP94, Xbp1, Chop (in both edited groups) ↑ Atf4 in Ldlr + Apob group Indicated ER stress from Apob loss Secondary Outcomes: Liver steatosis and ER stress in Ldlr + Apob gRNA group AAV-ITR integration events at cut sites detected One off-target site for Ldlr gRNA No systemic toxicity or weight loss | |
| 20 | Kelsey E. Jarrett, 2019 (20) | 14 males, 9 females | n = 7-13 per groups | Yes | Yes | mice | 6 weeks | Atherosclerosis | LDLR | CRISPR-Cas9 (SaCas9) | AAV8 vector | 5 × 10¹¹ genome copies per mouse, single injection | Primary Outcomes: Editing Efficiency Ldlr editing (NGS): Males: 31.9 ± 4.7% Females: 33.1 ± 15.1% Vector genome detection: Males: higher AAV transduction than females Females: lower AAV-CRISPR genome copies → lower editing Off-target Analysis: 4 predicted off-target sites tested → no detectable indels above background Some mice showed AAV-ITR fragment and full vector insertions at the cut site Protein Knockdown (Liver LDLR): Western blot: Almost complete loss in males with AAV-CRISPR Females showed partial knockdown; AAV-hPCSK9 was more effective in females Plasma Lipids: Cholesterol after 20 weeks: Males: AAV-CRISPR: 1408 ± 473 mg/dL AAV-hPCSK9: 993 ± 481 mg/dL Ldlr-KO: 1966 ± 412 mg/dL Females: AAV-CRISPR: 751 ± 456 mg/dL AAV-hPCSK9: 1142 ± 155 mg/dL Ldlr-KO: 1171 ± 228 mg/dL Lipoprotein profile: All treated groups showed elevated VLDL, IDL, and LDL cholesterol Atherosclerosis Lesion Area (Aorta, Oil Red O staining): Males: AAV-CRISPR: 7.76 ± 4.58% AAV-PCSK9: 3.89 ± 3.91% Ldlr-KO: 13.1 ± 3.83% Females: AAV-CRISPR: 2.84 ± 3.36% AAV-PCSK9: 7.99 ± 1.48% Ldlr-KO: 9.46 ± 2.75% Secondary Outcomes: No off-target mutations at predicted loci Insertion of AAV vector genome observed at cut site No systemic toxicity or weight loss reported Sex-based difference in transduction efficiency and phenotype noted | |
| 21 | Kiran Musunuru, 2021(21) | n = 3-4 | n = 4 | Not mention | Not mention | NHP | Not mention | Hyperlipidemia | PCSK9 | Adenine base editor (ABE8.8) | Lipid nanoparticles | 1.0 to 3.0 mg/kg. | Primary Outcomes: Genome Editing Efficiency: Editing frequency in liver hepatocytes: ~63–66% at the PCSK9 target splice site. Minimal indels observed (~0.5%). PCSK9 Protein Levels: Reduction in serum PCSK9 levels by ~90%. Sustained effect for 8 months post-treatment. Serum LDL Cholesterol: Reduction by 60% compared to baseline. Effect maintained for 8 months. Secondary Outcomes: Lipid Profiles: Additional reduction in lipoprotein(a) levels by 35%. Liver Function: Transient increase in liver enzymes (AST, ALT) during the first week. Liver enzymes normalized within 2 weeks. Off-Target Effects: Whole-genome sequencing and ONE-seq showed low-level off-target editing at one site (C5) in cynomolgus monkeys. No significant off-target editing in primary human hepatocytes. | |
| 22 | Lei Huang, 2017 (22) | n = 6 | n = 6 | Not mention | Not mention | pigs | Embryos | Hyperlipidemia | ApoE and LDLR | CRISPR-Cas9 | Electroporated into **embryonic** | Single embryo transfer for pig generation | Primary Outcomes: Serum Lipid Levels: Total Cholesterol (TC): ApoE-/-/LDLR-/- pigs: 3.05 ± 0.12 mM WT pigs: 1.94 ± 0.16 mM (P=0.0002) Triglycerides (TG): ApoE-/-/LDLR-/- pigs: 0.77 ± 0.08 mM WT pigs: 0.35 ± 0.06 mM (P=0.0015) Low-Density Lipoprotein Cholesterol (LDL-C): ApoE-/-/LDLR-/- pigs: 1.96 ± 0.06 mM WT pigs: 1.39 ± 0.13 mM (P=0.0030) Apolipoprotein B (APOB): ApoE-/-/LDLR-/- pigs: 186.21 ± 6.15 μg/mL WT pigs: 144.37 ± 12.56 μg/mL (P=0.0115) Long-term Lipid Elevation: Elevated TC, LDL-C, and HDL-C levels persisted in 12-month-old ApoE-/-/LDLR-/- pigs (P<0.05). Phenotypic Validation: The double knockout successfully induced early-onset dyslipidemia, resembling familial hypercholesterolemia in humans. Secondary Outcomes: Genetic Validation: PCR and sequencing confirmed biallelic mutations of ApoE and LDLR genes. Off-Target Analysis: No off-target mutations were detected in 28 predicted loci. | |
| 23 | Li Xu, 2019 (23) | n= 5 | n= 5 | Not mention | Yes | mice | postnatal day 3; | Duchenne Muscular Dystrophy (DMD) | DMD | CRISPR-Cas10 | AAVrh.74 v | 1 × 10¹² vg per mouse | Primary Outcomes: Gene Editing & Dystrophin Expression: Deletion of exons 21–23 restored the reading frame 11.1% ± 0.7% of cardiomyocytes expressed dystrophin (immunofluorescence) Western blot: 2.16% ± 0.37% of WT dystrophin levels in heart Highest AAV vector genome copy detected in heart Cardiac Function & Structure: Echocardiography (19 months): ↑ Cardiac output and stroke volume vs untreated mdx mice Histology: ↓ Fibrotic area (Masson's trichrome) ↓ Collagen content (Sirius red staining) ↓ serum cardiac troponin I Precision & Off-Target Editing: Deep sequencing: 76.3% precise ligation between DSBs (amplicon C) Small insertions (14.3%) and deletions (9.3%) No large deletions detected (PCR) No tumor development in treated animals; 2/5 untreated mdx mice developed spontaneous rhabdomyosarcoma Secondary Outcomes: Adverse Outcomes: Systemic Toxicity & Immune Response: No increased ALT, AST, or BUN levels in treated vs WT No histological abnormalities in liver or major organs No antibody response against SaCas9 in neonatally injected mice Mild anti-AAV immune response detected Adverse Outcomes No adverse outcomes in AAV-CRISPR–treated mice over 19 months No off-target-induced tumors or organ damage Untreated mdx mice more likely to develop spontaneous tumors with age | |
| 24 | Lili Wang, 2021 (24) | n = 10 | None specified | Not mention | Not mention | NHP | 3.9 – 5.8 years old | Hypercholesterolemia | PCSK9 | Engineered meganucleases (M1PCSK9 and M2PCSK9) | AAV8 or AAV3B | Single IV infusion from 2.0E+12 to 3.0E+13 GC/kg | Primary Outcomes: Editing Efficiency (On-target Indels in Liver) Range: 9.5% – 64.4%, depending on dose and vector type Stable for up to 3 years Editing confirmed via AMP-seq and amplicon-seq from serial liver biopsies PCSK9 and LDL-c Reduction Serum PCSK9 levels reduced to 15% – 83% of baseline LDL-c levels reduced to 44% – 85% of baseline Effects were sustained for 3 years post-treatment Example: RA1866 (3.0E+13 GC/kg): PCSK9 = 15.5%, LDL = 43.8% RA3169 (6.0E+12 GC/kg + prednisolone): PCSK9 = 20.2%, LDL = 44.4% Off-target Effects Off-target (OT) sites detected by: ITR-seq and amplicon-seq OT editing frequencies were low and stable, with most animals having minimal or no significant OT effects over 3 years Safety and Histopathology Transient transaminase elevation (ALT) in early phase Minimal-to-mild liver mononuclear cell infiltrates; considered background findings in NHPs Capsular fibrosis noted but likely due to repeated liver biopsies No evidence of long-term toxicity or immune pathology Secondary Outcomes: No systemic toxicity or significant health issues Transient immune responses to AAV capsid or meganuclease in some animals One animal (RA2125) euthanized due to false-positive TB skin test, not due to gene editing | |
| 25 | Lingmin Zhang, 2019 (25) | n = 6 per group | n = 6 per group | Not mention | Not mention | mice | Not mention | Hypercholesterolemia | PCSK10 | CRISPR/Cas9 | Gal-LGCP | Single administration with sgPcsk9 at 150 nM (in vitro) | Primary Outcomes: Gene Editing Efficiency (in vitro): Up to approximately 60% gene editing efficiency of Pcsk9 gene in Hepa 1-6 cells (verified by T7E1 assay and high-throughput sequencing). Gene Editing Efficiency (in vivo): Successful gene editing (Indels confirmed by deep sequencing) in liver tissues from treated mice. Serum PCSK9 protein levels significantly decreased in treated mice compared to controls (688.83 pg/mL vs significantly higher levels in controls). Plasma LDL-cholesterol significantly reduced by ~30% in treated group compared to controls (saline). Secondary Outcomes: Off-target Analysis: No detectable off-target mutagenesis at 10 potential off-target genomic sites analyzed by T7E1 assay and Sanger sequencing in both in vitro and in vivo experiments. Cytotoxicity: Gal-LGCP exhibited low cytotoxicity compared to Lipofectamine 2000 formulation in vitro (Hepa 1-6 cells). Biodistribution: Primarily targeted and accumulated in liver cells (82% of liver cells showed successful uptake). Minimal uptake observed in non-liver (nonparenchymal) cells (~1%). Histological Examination: Hematoxylin/Eosin and Masson’s staining of major organs indicated no observable histopathological abnormalities in liver and other organs. Biochemical Safety: No significant changes or adverse effects observed in HDL-cholesterol, alanine aminotransferase (ALT), aspartate aminotransferase (AST), and triglycerides levels among treated and control groups.g | |
| 26 | Lisa N. Kasiewic, 2023 (26) | Mice: n = 5 - 6 per group  NHPs: n = 6 - 10 | NHPs: n = 3 | Yes | Not mention | mice and NHP | Mice: 8–10 weeks NHPs: 2–3 years old | Hyperlipidemia | LDLR | Adenine base editor (ABE8.8) mRNA and guide RNA (gRNA) | GalNAc-Lipid Nanoparticles | Mice: 0.1–0.5 mg/kg, single injection NHPs: 2 mg/kg, single injection | Primary Outcomes: Editing Efficiency In LDLR-deficient NHPs: Standard LNPs: ~4.5% liver editing GalNAc-LNPs: 61% liver editing In WT NHPs: Standard LNPs: 58% GalNAc-LNPs: 64% In mice (Ldlr−/−): Up to 56% Angptl3 editing with GL6 GalNAc-LNPs Protein and Cholesterol Effects ANGPTL3 protein reduction: LDLR-KO NHPs: 89% WT NHPs: 90% (GalNAc-LNP) vs 75% (standard LNP) LDL-C (in LDLR-KO NHPs): Reduced by ~35% (~100 mg/dL) over 3 months LDLR knockout confirmation: ~68% genomic editing, 95% protein reduction, and >300 mg/dL LDL-C increase post-editing Tissue Distribution Editing restricted to liver Minimal off-target editing in other tissues (<2%). Secondary Outcomes: Transient increases in ALT/AST (normalized by day 14) Transient immune activation: TNF-α and MCP-1 returned to baseline within 7 days No long-term toxicity noted Well-tolerated in both WT and LDLR-deficient NHPs | |
| 27 | Luzi Yang, 2024 (27) | n = 3 per group | n = 3 per group | Not mention | Yes | mice | Postnatal day 1 and 5 weeks age | Myocardial infarction-induced cardiac dysfunction | CAMK2d | Adenine Base Editor | AAV9 | 5×10¹⁰ vg/g Subcutaneous injection and Intravenous injection | Primary Outcomes: Gene Editing Efficiency (Cardiac): Significant protein depletion (validated by Western blotting) of CaMKIIδ in the heart. Effective gene editing confirmed by next-generation sequencing (indels observed at target loci in heart). Specificity Enhancement with miR122TS: Reduction of liver leakage: >90% decrease in transgene-positive liver cells upon miR122TS incorporation. Reduced hepatic editing: miR122TS dramatically reduced unintended liver gene editing without impairing cardiac-specific editing efficiency.  Myocardial Infarction Model: Echocardiography confirmed that ABE-mediated CaMKIIδ gene editing significantly alleviated myocardial infarction-induced cardiac dysfunction. miR122TS did not negatively affect therapeutic cardiac benefits. Secondary Outcomes: Off-target Analysis: Explicit off-target gene editing was not reported; focus was primarily on the specificity between heart and liver. Cytotoxicity and Hepatic Safety: miR122TS incorporation did not elevate liver injury biomarkers (alanine aminotransferase [ALT] and aspartate aminotransferase [AST]) levels, suggesting good hepatic safety profile. AAV Integration: miR122TS effectively prevented ectopic AAV integration into liver genomic loci targeted by sgRNA-induced double-strand breaks, as shown by quantitative PCR. | |
| 28 | Luzi Yang, 2024 (28) | n = 3 per group | n = 3 per group | Not mention | Not mention | | Postnatal day 1 | Dilated cardiomyopathy | LMNA | Adenine Base Editor | Dual AAV system | Single administration with 2 × 10¹¹ vg/g | Primary Outcomes: Editing Efficiency: Up to ~20% at bystander site (c.1619T) with non-optimized sgRNAs ~8% at the disease-causing site (c.1621T) with optimized TadA8e-NG combination ~2.5%–5% with all-in-one AAV system Specificity: sgRNA1 reduced bystander editing Molecular Correction: Correction of Lmna c.1621C>T mutation Orthogonal editing of Camk2d (a therapeutic target for heart diseases) confirmed. Secondary Outcomes: No explicit report of: Off-target genome-wide sequencing (but computational prediction with DeepABE was used) Cardiac or systemic adverse effects | |
| 29 | Man Qi, 2024 (29) | n=8-10 per group | n=8-10 per group | Not mention | Not mention | mice | postnatal day 14 | Type 3 Long QT Syndrome | Scn5a | Adenine Base Editor (ABEmax) | Dual AAV9 vectors | 3×10¹⁴ GC/kg | Primary Outcomes: Editing Efficiency: DNA editing efficiency: 43.04% (homozygous), 37.72% (heterozygous). mRNA correction: Up to 99.20%. QT Interval: QTc prolongation in T1307M mice reversed to WT levels with >60% mRNA correction. QT/QTc interval improvement correlated with mRNA correction rates. Arrhythmia Prevention: Carbachol-induced arrhythmia: Sinus arrest significantly reduced. No Torsades de Pointes (TdP) or ventricular tachycardia (VT) observed in ABE-treated mice. Therapeutic Threshold: Correction rates >60% eliminated QT prolongation and arrhythmias. Secondary Outcomes: Body Weight: Restored in homozygous T1307M mice after ABE treatment. Cardiac Function: Action potential duration (APD90) normalized. Late sodium current (INa-L) reduced. Off-Target Effects: gRNA-dependent off-target editing: <1% at 30 predicted loci. Transcriptome-wide RNA sequencing: No significant A-to-G conversions No tumor formation: No liver or heart tumors after 17 weeks . | |
| 30 | Marco De Giorgi, 2021 (30) | n = 5–8 per groups | n = 5 | Yes | Not mention | mice | Adults: ~8 weeks Neonatal: Postnatal day 4 (P4) | Hypercholesterolemia  Hereditary Tyrosinemia Type I | ApoA1 | CRISPR-Cas9 (SaCas9) | Dual AAV8 system: | 5 × 10¹¹ GC each vector (total 1 × 10¹² GC) in adults 5 × 10¹¹ GC or dose-range (10¹² to 2.5 × 10¹⁰ GC) in neonates Single dose | Primary Outcomes: Editing Efficiency Indels at Apoa1 3′ UTR: Adults: ~54% HDR-mediated insertion: Adults: 1.8% (CRISPR+Donor); 0.3% (Donor only) Neonates: 7.8% (CRISPR+Donor); 0.1% (Donor only) NHEJ insertion (ITR-based): Adults: ~12% Neonates: ~21.5% Transgene Expression mKate2 expression (FLAG+ hepatocytes): Adults: ~6% of hepatocytes Neonates: ~16% of hepatocytes Functional Protein Output FIX: CRISPR+Donor: ~230 ng/mL Donor only: ~140 ng/mL APOE: Detected only in CRISPR+Donor mice Resulted in ↓ total cholesterol and triglycerides in Apoe−/− mice Cholesterol: 638 ± 58.7 mg/dL (vs. control ~1325 mg/dL) Triglycerides: 83.4 ± 7.8 mg/dL (vs. control ~155.7 mg/dL) FAH (HT-I model): Restored liver function, survival, FAH protein (~50% of WT) Corrected liver histology Secondary Outcomes: No liver toxicity, no tumor formation, or changes in plasma apoA1 No off-target activity at top 13 predicted sites AAV genome integration via NHEJ observed but well characterized Immunohistochemistry and Western blot confirmed accurate tissue targetingng | |
| 31 | Markus Grosch, 2022 (31) | n =2-5 per group | n = 4-5 per group | Not mentioned | Not mentioned | mice | 4 weeks | Dilated cardiomyopathy | RBM20 | Adenine Base Editor (ABE) | AAVMYO | tail vein injection 1 × 10¹² vector genomes (vg) | Primary outcomes: Editing Efficiency DNA editing in heart: up to 21.4% at 6 weeks, 18–20% at 12 weeks RNA editing (Rbm20 mRNA) in heart: up to 71% at 12 weeks Highest editing in heart > diaphragm > quadriceps; negligible in liver Molecular Correction Restoration of RBM20 nuclear localization in ~75% of cardiomyocytes Splicing rescue of RBM20 targets: TTN, Ldb3, Ryr2, Camk2d Protein: TTN isoform normalization (↓ G-TTN, ↑ N2B/N2BA isoforms) RNA-seq: 50% of mis-spliced exons corrected post-treatment Functional Rescue Echocardiography at 12 weeks post-injection: Ejection fraction (EF) nearly normalized to wild-type levels Decreased LVID and cardiac volume ↓ Nppa and Nppb (heart failure biomarkers) Transcriptomic Recovery snRNA-seq: restoration of transcriptional profile in cardiomyocytes Gene expression normalization observed in fibroblasts, endothelial cells, pericytes, myeloid cells, etc. Secondary outcomes: Adverse Outcomes Whole-genome sequencing (WGS): No A>G off-target mutations in heart No gRNA-homologous regions detected near variant sites RNA-seq: Slight (2%) increase in A>G mutations in 8e-NRCH-treated mice only Bystander edits: Present in some combinations, especially 8e-NRCH (~4%) Mostly synonymous or non-consequential mutations No evidence of immune activation, liver toxicity, or fibrosis | |
| 32 | Mengmeng Guo 2020 (32) | n =6 per group | n = 6 per group | Not mentioned | Not mentioned | hamsters | Embryos | Hypercholesterolemia | ApoC3 | CRISPR/Cas9 | zygote microinjection | | | Primary outcomes: Editing Efficiency Lipid Profile on Chow Diet: Significant reduction in triglyceride (TG) levels No change in total cholesterol (TC) and HDL-C Reduced VLDL fraction, increased LDL fraction (by lipoprotein disc electrophoresis) Lipid Profile on High-Fat/Cholesterol Diet: Significant reduction in TG and TC levels Significant increase in HDL-C Substantial reduction in VLDL and LDL fractions; increase in HDL Lower ApoB and ApoE levels; elevated ApoA1 levels (Western blot) Atherosclerosis Assessment: Significant reduction of atherosclerotic lesions in thoracic and abdominal arteries No statistical difference in lesions in the aortic arch and sinus Secondary outcomes: Safety and Physiological Effects: No reported adverse effects All procedures approved by the Animal Ethics Committee at Peking University (protocol LA2010-059) |
| 33 | Min Qiu, 2021 (33) | n = 5 per groups | n = 5 | Not mention | Not mention | mice | 6–8 weeks | Hypercholesterolemia | ANGPTL3 | CRISPR-Cas9 (SpCas9) | Lipid Nanoparticles | Single dose of 1.0, 2.0, or 3.0 mg/kg total RNA | Primary Outcomes: Editing Efficiency Median genome editing efficiency in liver: 38.5% with 306-O12B LNP (vs 14.6% with MC-3 LNP) Durable for at least 100–150 days post-injection Most common indels: 1-nt deletions and insertions at predicted Cas9 cut site Protein and Lipid Reduction Day 7 post-treatment (3.0 mg/kg): ANGPTL3 protein: ↓ 65.2% LDL-C: ↓ 56.8% TG: ↓ 29.4% Day 100 post-treatment: ANGPTL3: ↓ 60% LDL-C: ↓ 48.1% TG: ↓ 28.6% Off-target and Safety Off-target: NGS analysis of top 9 predicted sites → no detectable off-target editing Toxicity: ALT, AST, TNF-α normal at 48h and 100d Transient increase in IFN-α, IL-6, IP-10 at 6h, resolved by 48h No liver histological abnormalities or systemic toxicity Secondary Outcomes: No long-term toxicity, no sustained inflammatory response | |
| 34 | Ping Yang, 2024 (34) | n = 6–8 per group | n = 6–8 per group | Yes | Yes | mice | postnatal day 3 | Hypertrophic cardiomyopathy | MYH6 | CRISPR-Cas13d | AAV9 | 1×10¹¹ vg/mouse | Primary Outcomes: Gene Editing Efficiency: Allele-Specific Suppression: Myh6^R872H transcript knockdown: 27.1%. Myh6^R404Q transcript knockdown: 32%. Cardiac Phenotype: Myh6^R872H/+: Normalized left ventricular hypertrophy and ejection fraction. Reduced cardiomyocyte size and fibrosis. Myh6^R872H/R404Q: Significant prevention of ventricular hypertrophy. Improved ejection fraction and reduced arrhythmia. Arrhythmia Prevention: QTc prolongation, QRS complex, and PR interval abnormalities were normalized. Secondary Outcomes: Histological Analysis: Reduced cardiac wall thickening. Decreased cardiomyocyte cross-sectional area. Reduced fibrosis (by 43%) and cellular apoptosis. Off-Target Effects: Minimal off-target cleavage (<1%) detected at 30 loci. Reduced collateral cleavage activity compared to wild-type Cas13d. Comparable to “high-fidelity” hfCas13d, but with superior on-target activity | |
| 35 | Qian Li, 2021 (35) | n= 36 | n = 5 | Not mention | Not mention | mice | 4–6 weeks | Hypercholesterolemia | PCSK9 | CRISPR-Cas9 (SaCas9) | AAV8 vector | Single **IV tail vein injection** 2 × 10¹¹ genome copies per mouse | Primary Outcomes: On-Target Editing Efficiency (liver, SURVEYOR assay) Editing frequency: 25%–45% at 1, 8, and 24 weeks Similar to control (non-self-cleaving AAV-Cas9), showing no compromise in efficacy PCSK9 and Cholesterol Levels Serum PCSK9: ↓ ~80% Total cholesterol: ↓ ~35% Effects persisted up to 24 weeks Cas9 Protein and Viral Genome Copy Cas9 protein: ↓ ~60% by week 24 AAV genome copy: ↓ ~70% (suggesting self-cleavage mechanism reduces viral persistence) Off-Target and Immunogenicity Off-target mutations: Deep sequencing of top 10 predicted OT sites → 20-fold reduction in off-target activity with self-cleaving AAV vs. control Immune response: Neutralizing antibody (nAb) against SaCas9: significantly lower in self-cleaving group AAV8 capsid nAb: no significant difference AAV Integration Detected full-length and truncated AAV integration at PCSK9 locus in all treated animals ~30% of indels occurred in AAV vector genome at cleavage site Secondary Outcomes: Liver histology (H&E): no inflammation or damage AST/ALT: no significant changes No toxicity reported | |
| 36 | Qiang Cheng, 2020 (36) | n = 3 – 4 per group | n = 3 | Yes | Not mention | mice | 6–8 weeks | Hypercholesterolemia | PCSK9  PTEN Tomato reporter | Cas9 mRNA + sgRNA, or Cas9 RNP complexes | Lipid nanoparticles | Cas9 mRNA + sgRNA: 2.5 mg/kg, single do Cas9 RNP: 1.5 mg/kg, single dose PCSK9 study: 3 doses on Day 0, 2, and 4 | Primary Outcomes: On-Target Editing Efficiency (liver, SURVEYOR assay) Editing Efficiency tdTomato model (reporter gene editing): Liver: ~93% of hepatocytes Lung: 40% epithelial, 65% endothelial, 20% immune cells Spleen: 12% B cells, 10% T cells, 20% macrophages PTEN editing (T7E1 assay + TIDE): Liver: ~14% indels (20% DODAP SORT) Lung: ~15% indels (50% DOTAP SORT) RNP-based editing: 2.7% (liver), 5.3% (lung) PCSK9 Editing Indel rate at PCSK9: ~60% (TIDE analysis) Protein knockout: ~100% reduction in liver PCSK9 ~100% reduction in serum PCSK9 Phenotype: increased liver:body weight ratio (due to lipid accumulation) Secondary Outcomes: No abnormal AST, ALT, BUN, or creatinine levels No inflammatory cytokines (TNF-α, IL-1β) elevation No histopathological damage in liver, spleen, or lung tissues No immune-related adverse effects or organ damage with tested doses | |
| 37 | Qiurong Ding, 2014 (37) | n=5 per group | n=5 per groups | Not mention | Not mention | mice | 5 - 11 weeks | Hyperlipidemia | PCSK9 | CRISPR-Cas9 (SpCas9) | adenovirus | Single Intravenous administration. | Primary Outcomes: Genome Editing Efficiency: Rapid and efficient editing observed within 3–4 days Mutagenesis: Up to 50% of Pcsk9 alleles disrupted in hepatocytes. PCSK9 Protein Levels: Plasma PCSK9 reduced from ~26,461 pg/mL to 2,597 pg/mL (90% reduction). Serum Cholesterol Levels: Plasma total cholesterol reduced by 35%–40%: Treated group: 101 mg/dL. GFP group: 157 mg/dL. No virus group: 161 mg/dL. Liver LDLR Protein: Hepatic LDL receptor protein levels significantly increased in CRISPR-Pcsk9 treated mice. Secondary Outcomes: Lipoprotein Profile: HDL and LDL cholesterol fractions reduced in CRISPR-Pcsk9 treated mice. Tissue Safety: ALT levels: No significant difference between treated and control groups. Histology: No inflammation or tissue damage observed in liver sections. Off-Target Effects: No significant off-target mutagenesis detected at the 10 closest predicted loci. No liver toxicity or systemic inflammation | |
| 38 | Richard G. Lee, 2023 (38) | NHP: n = 4 - 22 per group Mice: n = 90 | n=10 | Not mention | Not mention | mice and NHP | Monkeys: Mean age ~29 months Mice: ~9 weeks | Hyperlipidemia | PCSK9 | Adenine Base Editor (ABE8.8m) | Lipid nanoparticles | Single intravenous infusion at **0.75 mg/kg** or **1.5 mg/kg** | Primary Outcomes: PCSK9 Editing Efficiency: Liver Editing: 0.75 mg/kg: 46% editing. 1.5 mg/kg: 70% editing. Serum PCSK9 Protein Levels: 0.75 mg/kg: 67% reduction. 1.5 mg/kg: 83% reduction. Serum LDL-C Levels: 0.75 mg/kg: 49% reduction. 1.5 mg/kg: 69% reduction. Durability: Effects sustained for up to 476 days post-treatment. Secondary Outcomes: Liver Safety: Transient increases in ALT (up to 572 U/L) and AST post-infusion. Resolved within 14 days without total bilirubin changes or liver damage. Histology: No macroscopic or microscopic liver abnormalities at 1-year necropsy. Germline Editing: Male Monkeys: No PCSK9 edits detected in sperm DNA. Mouse Offspring: 0 of 436 pups had germline transmission of the edit. | |
| 39 | Rui Lu, 2018 (39) | n= 7 | n = 4 | Not mention | Not mention | rabbits | Embryos | Hypercholesterolemia | LDLR | CRISPR-Cas9 | Microinjection | ingle microinjection per embryo | Primary Outcomes: Lipid and Lipoprotein Profiles at 12 Weeks Total Cholesterol, LDL-c: high ApoB and ApoE: ↑  ApoA-I: ↓ Lipoprotein types elevated: VLDL, LDL, chylomicron remnantsn) Atherosclerosis and Pathology Lesion area (Sudan IV staining): Different degrees of aortic lesions L9♀: 60% aortic lesion L13♂: 4% L17♀: 8% WT: 0% Histology: Macrophage-rich fibrous plaques, foam cells, calcifications Coronary artery atherosclerosis in L9♀ (HE staining) Xanthoma: observed in L10♀ paws Plasma inflammation markers (IL-1β, IL-6, CCL2): mostly not elevated Secondary Outcomes: No visible defects or reproductive issues No observed toxicity or mortality due to editing Plasma WBC counts and inflammatory markers largely normal at 48 weeks 6/13 edited pups died due to accidents, not genetic defects | |
| 40 | Samagya Banskota, 2022 (40) | n = 8 | n = 8 | Not mention | Not mention | mice | 6–8 weeks | Hyperlipidemia | PCSK9 | Adenine Base Editor | Engineered virus-like particles | Single systemic intravenous injection at **7 × 10¹¹** | Primary Outcomes: Editing Efficiency: Liver Editing: 63% adenine base editing in hepatocytes at the Pcsk9 locus. Serum PCSK9 Protein Levels: Reduced by 78% (compared to control). Serum Cholesterol Levels: Total serum cholesterol reduced by 40% compared to control mice. Phenotypic Validation: Therapeutic reduction in serum cholesterol levels confirmed post-editing. Secondary Outcomes: Off-Target Effects: No significant off-target editing detected using orthogonal assays. Biodistribution: Editing limited primarily to liver tissue. Safety: No signs of liver toxicity (ALT/AST levels remained normal). Histological examination showed no structural abnormalities in the liver. No glucose dysregulation or unexpected toxicity No germline editing | |
| 41 | Shijie Liu, 2021 (41) | n = 11 | n = 7 | Not mention | Yes | pigs | ~3 months | Cardiac Regeneration Post-Ischemia/Reperfusion-induced Myocardial Infarction | Sav | shRNA knockdown | AAV9 | subendocardial injection Low dose: 1 × 10¹³ vg total (≈ 3.4 × 10¹¹ vg/kg) High dose: 4 × 10¹³ vg total | Primary Outcomes: Editing and Target Validation Increased nuclear Yap localization in cardiomyocytes confirms Hippo pathway knockdown Effect observed in both GFP-positive and GFP-negative cells, suggesting paracrine signaling or division-induced dilution Cardiomyocyte Proliferation (in healthy and infarcted pigs) EdU incorporation (DNA synthesis marker): 3–4× more EdU+ CMs in treated hearts vs. control High-dose group: ~2.5% EdU+ CMs vs. 0.2% in control pHH3+ cells (M-phase marker): increased in treated hearts Aurora B kinase staining: presence of cytokinesis markers Higher mononucleation and CM density in treated groups Cardiac Function Improvement in LVEF: Control (AAV9-GFP): EF decreased by 6.3% Low dose: EF ↑ by 4.1% High dose: EF ↑ by 8.0% Improved stroke volume and reduced left ventricular end-systolic volume Reduced scar size (P = 0.0346) on infarct mapping Improved capillary density (P = 0.0159) Secondary Outcomes: No mortality in treated groups No tumors or abnormal histology in lung, liver, or kidneys No increase in systemic inflammation (CD45+ leukocytes normal) One pig (P-1946) died shortly after injection (likely mechanical complication) Mild injection-site inflammation observed in some animals but not clinically significant | |
| 42 | Shuhong Ma, 2021 (42) | n=5-8 per group | n=5-8 per group | Yes | Yes | mice | Embryos | Hypertrophic Cardiomyopathy | MYH6 R404Q/+ | ABEmax-NG | Microinjection: single-dose per embryo and Dual AAV9 vectors | Microinjection: single-dose per embryo AAV9: 7 μL each of AAV9.ABEmax-NG-N and AAV9.ABEmax-NG-C, single administration | Primary Outcomes: Editing Efficiency: Embryonic correction of R404Q allele: 62.5% correction in heterozygous embryos 70.8% correction rate in founder mice In utero AAV9 delivery: 25.3% correction of Myh6 mutation in heart tissue (DNA level) 19.6% correction in Fam178b (off-site locus used for comparison) Cardiac Functional Recovery: Echocardiography showed normalization of: Left ventricular wall thickness (LVWT) Ejection fraction (EF%) Fractional shortening (FS%) Cardiac hypertrophy (LVW/BW ratio) reduced Fibrosis area and cardiomyocyte cross-sectional area normalized Molecular and Transcriptomic Outcomes: RNA-Seq confirmed: Downregulation of hypertrophic genes (Nppa, Nppb, Myh7) Restoration of energy metabolism and ECM remodeling gene expression profiles Metabolic Analyses: Oxygen consumption rates and mitochondrial respiration parameters improved in edited cardiomyocytes. Secondary Outcomes: Adverse Outcomes: No detectable off-target edits at 989 predicted sites (DNA or RNA) No developmental or survival defects in founder mice A-to-I RNA edits seen transiently in early embryos but absent in adult mice No indels or insertions detected (vs. CRISPR/ssODN group with >50% indels) Low off-target mutations (<1%) detected at predicted loci using deep sequencing. | |
| 43 | Shuo Wu, 2024 (43) | n=6 per group | n=6 per group | Not mention | Not mention | mice | Postnatal days 0–3 | Hypertrophic cardiomyopathy | MYBPC3 | Base editing (SpRY-ABEmax and SpRY-ABE8e) | Dual AAV9 | Single subcutaneous injection Low dose: 0.5 × 10¹⁴ vg/kg and High dose: 1 × 10¹⁴ vg/kg | Primary Outcomes: Editing Efficiency: Low dose: ~4.6% A-to-G correction High dose: ~9.6% A-to-G correction, estimated ~30% in cardiomyocytes Protein Restoration: Low dose: 38%–70% MYBPC3 recovery High dose: 78%–110% MYBPC3 recovery (higher than gene correction rates, possibly due to improved protein stability) Cardiac Functional Outcomes: Significant improvement in ejection fraction (EF), ventricular size, wall thickness High dose preserved function up to 6 months; low dose and AAV-Mybpc3 effect waned over time Histological Improvement: High dose prevented cardiac enlargement, myofiber disarray, fibrosis All treatments reduced cardiomyocyte hypertrophy, but high dose had the strongest effect Molecular Outcomes (RNA-seq): Downregulation of heart failure and fibrosis markers (Acta1, Nppa, Col4a4) Restoration of cardiac development and conduction genes Transcriptome profile of treated hearts shifted toward wild-type pattern. Secondary Outcomes: Off-target DNA editing: Nearly background levels across 17 predicted sites (HT-seq) Off-target RNA editing: Minimal, comparable to saline controls Tissue specificity: Low editing in non-cardiac tissues (liver, lung, spleen, muscle) | |
| 44 | Simon Lebek, 2023 (44) | n=8 | n=8 per group | Not mention | Not mention | mice | 12 weeks | Ischemia/Reperfusion (IR) injury | CaMKIIδ (Camk2d) | ABE8e-SpRY | Dual AAV9 vectors | Direct intramyocardial injection: 7.5×10¹¹ vg/kg | Primary Outcomes: Editing Efficiency: DNA level (whole heart): ~7.5–8.4% for 3 target adenines cDNA level (whole heart): ~46% cDNA level (anterior wall): up to 85.7% at target sites Off-target editing: No off-target edits in CaMKIIα, β, or γ genes Minimal off-target A-to-G in DAZL intron (not expressed in heart) Cardiac Functional Recovery: Echocardiography and MRI showed: Recovery of fractional shortening to near sham levels at 3 weeks post-IR Prevention of LV dilation Reduced oxidized and autophosphorylated CaMKIIδ and RyR2 phosphorylation Improved calcium transients, reduced arrhythmias in iPSC-CMs Histopathology: Reduced apoptosis (TUNEL assay) Reduced fibrosis (trichrome stain) Reduced inflammation in myocardium Transcriptomics: 209 DEGs between IR+control and IR+edited groups Edited mice showed reversal of IR-induced upregulation of disease genes and restoration of cardiac function genes GO enrichment: restoration of cardiac muscle function and reduction in stress-response pathways Secondary Outcomes: Long-term safety: Mice injected with AAV-ABE-sgRNA6 at postnatal day 5 (P5) followed for 260 days No difference in body weight, cardiac function, or exercise capacity vs controls No increased off-target RNA A>I editing | |
| 45 | Simon Lebek, 2023 (45) | n=3-8 per group | n=8 | Yes | Yes | mice | 12 weeks | Cardiac Regeneration Post Ischemia/reperfusion (IR) injury | CAMK2D | Adenine base editor (ABE8e ) | MyoAAV2A | Single injection at **1.5×10¹¹ vg/kg** | Primary Outcomes: Editing Efficiency: sgRNA1: 36.2% (DNA); 83.2% (cDNA) for M281V. sgRNA2: 37.0%, 27.8%, and 36.4% for M281V, M282V, H283R respectively (DNA); ~83% at cDNA level. Cardiac Function: Fractional Shortening: Control virus: 40.3%. sgRNA1: 47.2% (P=0.09). sgRNA2: 51.6% (P=0.02). Left Ventricular End-Diastolic Diameter (LVIDd): Control virus: 3.3 mm. sgRNA2: 2.8 mm (P=0.01). Fibrosis Reduction: Control virus: 9.3% fibrotic area. sgRNA2: 1.8% fibrotic area (P<0.0001). Secondary Outcomes: Exercise Performance: Maximal Velocity: Control virus: 18 m/min. sgRNA1: 21.1 m/min. sgRNA2: 25.9 m/min (P<0.0001). Total Distance: Control virus: 109.2 m. sgRNA2: 405.4 m. Oxidized CaMKII: Control virus: ~4.7-fold increase. sgRNA2: Significantly reduced (P<0.0001). CaMKII Activity: Control virus: 8.9 nmol/min/mg. sgRNA2: 1.5 nmol/min/mg. Adverse Outcomes Off-target editing: No significant editing at top 8 predicted sites (sgRNA1) sgRNA2 reduced off-target DAZL editing from 29.5% to 9.2% Organ weights: No abnormal liver weight; heart and lung weights normalized in sgRNA2 group Liver toxicity: None observed Systemic effects: No signs of immune or adverse systemic reaction | |
| 46 | Simon Lebek, 2024 (46) | n=9 | n=26 | Yes | Not mention | mice | Embryos | Severe transverse aortic constriction (sTAC) to induce afterload-driven heart failure | CaMK2D (CaMKIIδ) | ABE8e fused to SpCas9-NG | Embryonic microinjection into zygotes | Not mention | Primary Outcomes: Survival: WT-sTAC: 65% mortality within 2 weeks post-surgery T287A-sTAC: only 11% mortality Cardiac function: WT-sTAC: fractional shortening dropped from ~60% to 14.3% ± 1.5% T287A-sTAC: 33.9% ± 6.5%, significantly better Less LV dilation and hypertrophy in T287A-sTAC vs. WT-sTAC Histology & pathology: WT-sTAC: increased fibrosis (1.8× vs. sham), cardiomyocyte apoptosis (15.7× vs. sham) T287A-sTAC: minimal fibrosis, apoptosis similar to sham Transcriptomics: RNA-seq: 5994 DEGs in WT-sTAC vs. sham (upregulated genes linked to remodeling/inflammation) 3787 DEGs in T287A-sTAC vs. WT-sTAC T287A-sTAC preserved expression of cardiac performance/metabolism genes Human iPSC-derived cardiomyocyte validation: ABE-edited iPSC-CMs (T287A): Resistant to chronic β-adrenergic stress (ISO-induced) Normal Ca²⁺ transients, less arrhythmia (6.5× reduction), improved calcium handling 2000× specificity for CaMKIIδ vs other isoforms (α, β, γ) No significant off-target edits (DNA sequencing of top 8 predicted sites) ). Secondary Outcomes: Adverse Outcomes No cardiac dysfunction in T287A mice under basal conditions No off-target edits detected in CaMKIIα/β/γ or top 8 predicted genomic loci No systemic toxicity observed | |
| 47 | Suya Wang, 2020 (47) | n = 10-12 | n 6-12 | Yes | Yes | mice | Neonatal (P1), young (P20), and adult (up to 6 months) | Barth Syndrome | TAZ | gene replacement | AAV9 | High dose: 2 × 10¹⁰ vg/g (~70% CM transduction) Medium dose: 1 × 10¹⁰ vg/g (~33% CM transduction) Single dose administration Subcutaneous injection (P1) Retro-orbital (P20 or older) | Primary Outcomes:  Survival and Growth AAV-hTAZ rescued neonatal lethality in low birth weight TAZ-KO mice Rescued mice showed normalized weight gain and improved survival  Cardiac Function Echocardiography: High-dose AAV-hTAZ: Normalized fractional shortening (FS%) Prevented LV dilation and cardiac hypertrophy Medium-dose: partial effects with variability Histology: ↓ fibrosis (Sirius red staining) ↓ CM apoptosis (TUNEL assay) Cardiolipin profile: ↓ MLCL:CL ratio, normalized in high-dose group Molecular and Ultrastructural Correction Restoration of TAZ protein (capillary immunoblot) Normalization of mitochondrial morphology and gene expression (e.g., Opa1, Mfn2, Atp6) Improved mitochondrial cristae alignment and complexity (EM analysis) Skeletal Muscle & Exercise Capacity Partial rescue in skeletal muscle (↑ fiber area, improved mitochondrial area) Modest improvement in endurance (treadmill test, not statistically significant) Secondary Outcomes: No toxicities reported High AAV-hTAZ doses were safe and effective in both neonates and adults Therapeutic durability depended on CM transduction rate (≥ 70% required for sustained effect) | |
| 48 | Takahiko Nishiyama, 2022 (48) | n=8 | n=10 | Not mention | Yes | mice | postnatal 5 days | Dilated cardiomyopathy | RBM20R636Q/R636Q | Adenine base editor (ABEmax-VRQR-SpCas9) | AAV9 | single intraperitoneal injection 2.5 × 10¹⁴ vg/kg | Primary Outcomes: Editing Efficiency: DNA level editing efficiency in mouse hearts: 19%. RNA level (cDNA) editing: 66% correction. Cardiac Function: Fractional Shortening: Untreated: ~19%. ABE-treated: ~40% (normalized to wild-type levels). Left Ventricular Internal Diameter: End-diastolic (LVIDd): Normalized post-treatment. Survival: Untreated mice: Premature death by 2–3 months. ABE-treated: Extended survival (lifespan significantly prolonged). Rescue of Gene Splicing: Restoration of proper Ttn splicing (N2B isoform normalized to 68%). Secondary Outcomes: Histological Analysis: Reduced fibrosis and ventricular dilation in treated mice. Localization of RBM20: Restored nuclear localization and elimination of cytoplasmic RNP granules. Transcriptome Normalization: RNA-seq showed recovery of DCM-related transcriptional profiles. Inflammatory Response: Minimal immune cell infiltration post-AAV9 treatment. Adverse Outcomes Off-target DNA/RNA editing: None detected at top predicted sites Immunogenicity: Slight increase in cardiac immune cells (11.2% vs 9.8%) Tumorigenesis: No liver tumors observed up to 3 months No systemic toxicity: Cardiac-specific expression limited adverse effects | |
| 49 | Tanja Rothgangl, 2021 (49) | Mice: n = 3 per group NHP: n = 12 | n = 8 | Not mention | Not mention | mice | Adult mice (6–8 weeks) and macaques (~3 years old) | Hyperlipidemia | PCSK9 | Adenine Base Editor (ABEmax ) | Lipid nanoparticles | Single intravenous injection at **1–2 mg/kg** | Primary Outcomes: Editing Efficiency: Mice: Editing efficiency at Pcsk9: 58% in hepatocytes. Macaques: Editing efficiency at PCSK9: 35–40%. PCSK9 Protein Levels: Mice: Plasma PCSK9 levels reduced by 85%. Macaques: Plasma PCSK9 levels reduced by 65–75%. Serum LDL-C Levels: Mice: Serum LDL-C levels reduced by 35–40%. Macaques: LDL-C levels reduced by 30–50% post-treatment. Secondary Outcomes: Biodistribution: Editing confined to liver tissues, no detectable off-target editing in other organs. Safety: Transient increase in liver enzymes (ALT/AST), normalized within 14 days. No significant immune response or adverse events observed. No tumor formation, Off-Target Effects: Whole-genome sequencing detected no significant off-target effects at predicted loci. | |
| 50 | Xiao Wang, 2017 (50) | n = 5 per group | n = 6 per group | Not mention | Not mention | mice | 5-month old age | hepatocytes engrafted mice | PCSK10 | CRISPR-Cas9 | Adenovirus | Single adenoviral injection | Primary Outcomes: On-target Editing Efficiency: ~47% and 42% indel rates by deep sequencing in two representative mice 75% of indels were 1–2 bp insertions or deletions at the expected cut site Functional Effects: ~52% reduction in blood human PCSK9 protein levels compared to pre-treatment No change in human albumin levels (engraftment marker) Mouse PCSK9 protein increased >2-fold post-treatment (compensatory response) No significant change in total cholesterol levels. Safety: Deep sequencing at 8 predicted off-target sites showed no detectable off-target indels above background error rates | |
| 51 | Xiaolu Pan, 2018 (51) | n = 6 | n = 7 | Yes | Yes | mice | postnatal 10 days | Catecholaminergic polymorphic ventricular tachycardia | RYR2 R176Q/+ | CRISPR-Cas9 (SaCas9 ) | AAV9 | ingle subcutaneous injection at **5×10¹¹ to 1×10¹² GC/mouse** | Primary Outcomes: Arrhythmia Susceptibility: Sustained VT (after isoproterenol and caffeine administration): Control group: 71% incidence. AAV9-CRISPR treated group: 0% incidence (P<0.05). Gene Editing Efficiency: Indel Frequency at Ryr2 mutant allele: DNA: 11.3% in treated R176Q/+ mice. RNA: 21.1% editing efficiency (due to nonsense-mediated decay). Calcium Spark Frequency (Ca²⁺ handling in myocytes): Untreated R176Q/+ group: 2.3 a.u. AAV9-CRISPR treated group: 1.4 a.u. (normalized, P<0.01). Secondary Outcomes: RyR2 Protein Levels: Total RyR2 protein reduced by 25–30% in treated R176Q/+ mice (P<0.05). Calcium Handling: SR Ca²⁺ transient amplitudes unaffected. Reduced inappropriate Ca²⁺ leak and normalization of SR Ca²⁺ sparks. Histological and Functional Safety: No effect on cardiac contractility (ejection fraction unchanged). Off-Target Effects: No detectable off-target indels at predicted loci (deep sequencing). Adverse Outcomes No arrhythmia in WT or control-treated mice AAV vector insertions occurred, but only in edited mutant allele No signs of inflammation or organ damage No impact on cardiac contractility in edited animals | |
| 52 | Xin Guo, 2017 (52) | n = 16 - 20 per group | ≥16 wild-type | Not mention | Not mention | hamsters | 8–10 weeks (young adults); 12–18 months (aged) | Hypercholesterolemia | LDLR | CRISPR-Cas9 | Microinjection | Single embryo injection | Primary Outcomes: Genotyping and Knockout Validation Exon 2 targeted → founder lines with 10–194 bp deletions Western blot: LDLR undetectable in Ldlr−/−, ~50% in Ldlr+/− Plasma Lipid Levels (Chow Diet, 2 months) Ldlr−/− showed highest total cholesterol and triglycerides FPLC: Ldlr−/− showed high LDL + VLDL; phenotype similar to human FH HCHF Diet Challenge (12 Weeks) Ldlr+/−: TC ↑ >2000 mg/dL, TG ↑ >1200 mg/dL Aortic lesions: ↑ in whole aorta, arch, thoracic, abdominal regions Coronary lesions: 52% unaffected 26% minor (<5%) 21% partial (5–20%) occlusion Ldlr−/− Hamsters on HCHF TC up to 6000 mg/dL, severe hyperlipidemia 50% mortality by day 30 Advanced coronary atherosclerosis + myocardial fibrosis Lipid-Lowering Drug Response (Ldlr+/− on HCHF) Ezetimibe: most effective in lowering TC Fenofibrate, Rosuvastatin: moderate effect FPLC: reduction in LDL/VLDL fractions Secondary Outcomes: Ldlr−/− hamsters on HCHF showed early mortality, severe lesions, fibrosis No overt inflammation or organ toxicity in Ldlr+/− on drug treatment Embryo editing showed no off-target mutations detected | |
| 53 | Yiwen Zha, 2021 (53) | n = 3 | n = 5 | Not mention | Not mention | rabbits | Embryos | Hyperlipidemia and atherosclerosis | APOC3 | CRISPR-Cas9 | Microinjection | Microinjection into zygotes with Cas9 mRNA (33 ng/µL) and sgRNA (40 ng/µL) targeting exon 2 | Primary Outcomes: Lipid Profile – Normal Chow Diet KO rabbits had ~50% lower TG, slightly lower LDL-C, and slightly higher HDL-C Lipid Profile – HFD (12 Weeks) WT: TC ~805 mg/dL, TG ~891 mg/dL, LDL-C ~403 mg/dL, HDL-C ~25 mg/dL KO: TC ~384 mg/dL, TG ~276 mg/dL, LDL-C ~229 mg/dL, HDL-C ~38 mg/dL KO group had significantly reduced rise in all lipid parameters under HFD challenge Atherosclerosis Aortic lesion area: WT: 21% of aortic area KO: 3% Histology: WT: intimal thickening, macrophage infiltration, collagen, smooth muscle proliferation KO: mild early lesions only Coronary arteries: no significant lesions in either group Inflammation KO rabbits had ↓ IL-1β and TNF-α, ↓ monocyte, neutrophil, and platelet counts Histology showed fewer macrophages in plaques (RAM-11) Lipid Metabolism Enzymes ↑ Lipoprotein lipase (LPL) and hepatic lipase (HL) activities in KO group KO rabbits showed faster TG clearance in oral fat tolerance test (OFTT) Apolipoprotein Profile ↓ APOC3, APOB, APOE ↑ APOA1 (linked to ↑ HDL-C) Off-target Effects 5 predicted off-target sites tested → no mutations detected Secondary Outcomes: No toxicities or physiological abnormalities in KO rabbits 2/5 pups died at birth (likely unrelated to gene editing) Liver histology: WT rabbits had lipid droplet accumulation, KO rabbits had minimal damage | |
| 54 | Yu Zhang, 2020 (54) | n = 3-6 per group | n = 3-6 | Not mention | Not mention | mice | Postnatal | Duchenne Muscular Dystrophy | DMD | CRISPR-Cas9 | Dual AAV system | single intraperitoneal injection: 4 × 10¹², 1.6 × 10¹³, 8 × 10¹³ vg/kg | Primary Outcomes: Efficiency and Dystrophin Restoration scAAV outperformed ssAAV by 20–70× in efficiency Dystrophin expression (IHC + Western blot, 4 weeks post-injection): scAAV (1.6 × 10¹³ vg/kg): >50% in TA/triceps, >70% in diaphragm, ~100% in heart ssAAV (same dose): <18% in TA/triceps, ~30% in diaphragm cDNA editing: >60% of events were +1-nt insertions, restoring ORF Muscle Function Specific force of EDL (fast-twitch): scAAV high dose: ↑ to 82% of WT ssAAV high dose: ↑ to 66% Soleus (slow-twitch): scAAV high dose: ↑ to 96% of WT ssAAV high dose: ↑ to 85% CK levels: scAAV reduced serum CK by 87–95% ssAAV-treated mice still had 8.5–18.6× higher CK than WT Histological Findings scAAV drastically reduced % of myofibers with central nuclei (sign of regeneration/damage): <5% in scAAV vs >70% in ssAAV-treated at same dose Muscle structure in high-dose scAAV-treated mice resembled WT Secondary Outcomes: No evidence of toxicity or inflammatory responses reported No significant immune reaction noted in neonatal mice No tumorigenesis or off-target pathology described | |
| 55 | Yuanbojiao Zuo, 2023 (55) | n = 8 | n =6 - 8 per group | Not mention | Not mention | mice | 6 weeks | Hyperlipidemia and atherosclerosis | ANGPTL3 | CRISPR-Cas9 | Dual AAV9 vectors | Single tail vein injection 2 × 10¹⁴ vg/kg total dose | Primary Outcomes: Efficiency and Dystrophin Restoration Liver DNA editing: 63.3 ± 2.3% at the target locus Angptl3 transcript: reduced by 88.0 ± 0.8% Liver ANGPTL3 protein: undetectable by Western blot Editing was liver-specific, undetectable in heart, kidney, and skeletal muscle Plasma Protein and Lipid Levels ANGPTL3: ↓ from 161.2 ± 9.2 ng/mL to 8.3 ng/mL at 2–4 weeks Triglycerides (TG): ↓ from 19.3 ± 1.5 mg/dL to 8.1 ± 0.8 mg/dL (↓ ~58%) Total cholesterol (TC): ↓ ~61% at 4 weeks Off-Target Effects Bystander edits: C4 and C5 were also edited (significant but expected) Cas-OFFinder: identified 12 potential off-targets; two with 0 mismatches in critical region tested OT1 and OT2: low but detectable off-target editing in vitro (N2a cells) gRNA-independent editing: low but measurable editing shown in R-loop assay Secondary Outcomes: AST and ALT: unchanged post-AAV delivery (no liver toxicity) H&E staining: no liver inflammation or damage CD3+ T cell infiltration: not observed (no immune infiltration) No adverse outcomes reported | |
| 56 | Zhanzhao Liu, 2025 (56) | n = 4–5 per group | n = 4–5 per group | Not mention | Not mention | mice | Postnatal day 1 | silencing gene therapy | CAMK2d | Adenine Base Editors (SauriABE and SpCas9-ABE) | AAV9 and dual AAV | Single administration 1 × 10¹¹ vector | Primary Outcomes: Editing Efficiency: Best editing (~high rates, exact percentage not specified) achieved with sgRNA7 Editing was heart-specific due to chromatin accessibility; little editing in liver Molecular Effects: Increased intron7 retention → premature stop codon → nonsense-mediated decay Significant decrease in total Camk2d mRNA (RT-PCR, RNA-seq) Depletion of CaMKIIδ protein (Western blot) Single vs Dual AAV: Single AAV outperformed all dual AAV constructs at all doses tested Editing was more efficient in neonates compared to adults (due to chromatin accessibility differences) Secondary Outcomes: Off-target Effects: Not explicitly detailed in summary; assumed minimal based on sgRNA screening and cardiac specificity Tissue Specificity: Editing confined to heart; no meaningful editing in liver or skeletal muscle Epigenetic Considerations: Editing efficiency was lower in adult hearts, linked to reduced chromatin accessibility | |
| 57 | Zhiquan Liu, 2021 (57) | gene targeting: n = 4 mice Zygote injection experiments: ~15 zygotes | n=5–6 mice per group | Not mention | Not mention | mice | 8–10 weeksAAV delivery: 8-week-old Zygote injection: Embryonic stage | Hyperlipidemia | PCSK9, TYR, MSTN | CRISPR-Cas9 (SpaCas9) | AAV8 | Single tail-vein injection of **2 × 10¹¹ genome copies (GC)/mouse** | Primary Outcomes: Editing Efficiency Pcsk9 (in vivo AAV delivery): 16.6% average indel rate in liver tissue at Pcsk9 site Zygote editing (Tyr, Mstn): Editing efficiency in blastocysts: 40–78% Base editing with Spa-CBE: 51.1–72.0% (C-to-T) Base editing with Spa-ABE: 28.5–63.0% (A-to-G) F0 mice showed albino phenotype consistent with Tyr mutation 2/4 F0 pups showed successful editing Functional Outcomes Pcsk9-edited mice: ↓ serum cholesterol (significant reduction at Day 30 post-injection) No change in ALT or albumin levels (no hepatotoxicity)Secondary Outcomes: Liver Function: Serum ALT and albumin levels remained within normal ranges, indicating no liver toxicity. Adverse Outcomes Off-target mutations: Low off-targets (<1%) in Pcsk9-edited livers (by deep sequencing) No off-targets detected in edited Tyr F0 mice Liver toxicity: No increase in ALT, no liver damage detected Immune response: Not discussed Tumorigenesis: Not observed | |

**Included studies**

1. Carreras A, Pane LS, Nitsch R, Madeyski-Bengtson K, Porritt M, Akcakaya P, et al. In vivo genome and base editing of a human PCSK9 knock-in hypercholesterolemic mouse model. BMC Biol. 2019;17(1):4.

2. Chadwick AC, Evitt NH, Lv W, Musunuru K. Reduced Blood Lipid Levels With In Vivo CRISPR-Cas9 Base Editing of ANGPTL3. Circulation. 2018;137(9):975-7.

3. Chai AC, Cui M, Chemello F, Li H, Chen K, Tan W, et al. Base editing correction of hypertrophic cardiomyopathy in human cardiomyocytes and humanized mice. Nat Med. 2023;29(2):401-11.

4. Li B, Guo Y, Zhan Y, Zhou X, Li Y, Zhao C, et al. Cardiac Overexpression of XIN Prevents Dilated Cardiomyopathy Caused by TNNT2 ΔK210 Mutation. Front Cell Dev Biol. 2021;9:691749.

5. Breton C, Furmanak T, Avitto AN, Smith MK, Latshaw C, Yan H, et al. Increasing the Specificity of AAV-Based Gene Editing through Self-Targeting and Short-Promoter Strategies. Mol Ther. 2021;29(3):1047-56.

6. Long C, Amoasii L, Mireault AA, McAnally JR, Li H, Sanchez-Ortiz E, et al. Postnatal genome editing partially restores dystrophin expression in a mouse model of muscular dystrophy. Science. 2016;351(6271):400-3.

7. Reichart D, Newby GA, Wakimoto H, Lun M, Gorham JM, Curran JJ, et al. Efficient in vivo genome editing prevents hypertrophic cardiomyopathy in mice. Nat Med. 2023;29(2):412-21.

8. Ran FA, Cong L, Yan WX, Scott DA, Gootenberg JS, Kriz AJ, et al. In vivo genome editing using Staphylococcus aureus Cas9. Nature. 2015;520(7546):186-91.

9. Hu H, Wang L, Li H, Li H, Chen X, Peng W, et al. Long-term amelioration of an early-onset familial atrial fibrillation model with AAV-mediated in vivo gene therapy. Fundam Res. 2022;2(6):829-35.

10. Yin H, Song CQ, Suresh S, Wu Q, Walsh S, Rhym LH, et al. Structure-guided chemical modification of guide RNA enables potent non-viral in vivo genome editing. Nat Biotechnol. 2017;35(12):1179-87.

11. Du H, Zhao Y, Wen J, Dai B, Hu G, Zhou Y, et al. LncRNA DCRT Protects Against Dilated Cardiomyopathy by Preventing NDUFS2 Alternative Splicing by Binding to PTBP1. Circulation. 2024;150(13):1030-49.

12. Zhao H, Li Y, He L, Pu W, Yu W, Li Y, et al. In Vivo AAV-CRISPR/Cas9-Mediated Gene Editing Ameliorates Atherosclerosis in Familial Hypercholesterolemia. Circulation. 2020;141(1):67-79.

13. Dave J, Raad N, Mittal N, Zhang L, Fargnoli A, Oh JG, et al. Gene editing reverses arrhythmia susceptibility in humanized PLN-R14del mice: modelling a European cardiomyopathy with global impact. Cardiovasc Res. 2022;118(15):3140-50.

14. Davis JR, Wang X, Witte IP, Huang TP, Levy JM, Raguram A, et al. Efficient in vivo base editing via single adeno-associated viruses with size-optimized genomes encoding compact adenine base editors. Nat Biomed Eng. 2022;6(11):1272-83.

15. Li J, Zhu D, Hu S, Nie Y. CRISPR-CasRx knock-in mice for RNA degradation. Sci China Life Sci. 2022;65(11):2248-56.

16. Gong J, Wang HX, Lao YH, Hu H, Vatan N, Guo J, et al. A Versatile Nonviral Delivery System for Multiplex Gene-Editing in the Liver. Adv Mater. 2020;32(46):e2003537.

17. Finn JD, Smith AR, Patel MC, Shaw L, Youniss MR, van Heteren J, et al. A Single Administration of CRISPR/Cas9 Lipid Nanoparticles Achieves Robust and Persistent In Vivo Genome Editing. Cell Rep. 2018;22(9):2227-35.

18. Levy JM, Yeh WH, Pendse N, Davis JR, Hennessey E, Butcher R, et al. Cytosine and adenine base editing of the brain, liver, retina, heart and skeletal muscle of mice via adeno-associated viruses. Nat Biomed Eng. 2020;4(1):97-110.

19. Jarrett KE, Lee CM, Yeh YH, Hsu RH, Gupta R, Zhang M, et al. Somatic genome editing with CRISPR/Cas9 generates and corrects a metabolic disease. Sci Rep. 2017;7:44624.

20. Jarrett KE, Lee C, De Giorgi M, Hurley A, Gillard BK, Doerfler AM, et al. Somatic Editing of Ldlr With Adeno-Associated Viral-CRISPR Is an Efficient Tool for Atherosclerosis Research. Arterioscler Thromb Vasc Biol. 2018;38(9):1997-2006.

21. Musunuru K, Chadwick AC, Mizoguchi T, Garcia SP, DeNizio JE, Reiss CW, et al. In vivo CRISPR base editing of PCSK9 durably lowers cholesterol in primates. Nature. 2021;593(7859):429-34.

22. Huang L, Hua Z, Xiao H, Cheng Y, Xu K, Gao Q, et al. CRISPR/Cas9-mediated ApoE-/- and LDLR-/- double gene knockout in pigs elevates serum LDL-C and TC levels. Oncotarget. 2017;8(23):37751-60.

23. Xu L, Lau YS, Gao Y, Li H, Han R. Life-Long AAV-Mediated CRISPR Genome Editing in Dystrophic Heart Improves Cardiomyopathy without Causing Serious Lesions in mdx Mice. Mol Ther. 2019;27(8):1407-14.

24. Wang L, Breton C, Warzecha CC, Bell P, Yan H, He Z, et al. Long-term stable reduction of low-density lipoprotein in nonhuman primates following in vivo genome editing of PCSK9. Mol Ther. 2021;29(6):2019-29.

25. Zhang L, Wang L, Xie Y, Wang P, Deng S, Qin A, et al. Triple-Targeting Delivery of CRISPR/Cas9 To Reduce the Risk of Cardiovascular Diseases. Angew Chem Int Ed Engl. 2019;58(36):12404-8.

26. Kasiewicz LN, Biswas S, Beach A, Ren H, Dutta C, Mazzola AM, et al. GalNAc-Lipid nanoparticles enable non-LDLR dependent hepatic delivery of a CRISPR base editing therapy. Nat Commun. 2023;14(1):2776.

27. Yang L, Liu Z, Chen G, Chen Z, Guo C, Ji X, et al. MicroRNA-122-Mediated Liver Detargeting Enhances the Tissue Specificity of Cardiac Genome Editing. Circulation. 2024;149(22):1778-81.

28. Yang L, Liu Z, Sun J, Chen Z, Gao F, Guo Y. Adenine base editor-based correction of the cardiac pathogenic Lmna c.1621C > T mutation in murine hearts. J Cell Mol Med. 2024;28(4):e18145.

29. Qi M, Ma S, Liu J, Liu X, Wei J, Lu WJ, et al. In Vivo Base Editing of Scn5a Rescues Type 3 Long QT Syndrome in Mice. Circulation. 2024;149(4):317-29.

30. De Giorgi M, Li A, Hurley A, Barzi M, Doerfler AM, Cherayil NA, et al. Targeting the Apoa1 locus for liver-directed gene therapy. Mol Ther Methods Clin Dev. 2021;21:656-69.

31. Grosch M, Schraft L, Chan A, Küchenhoff L, Rapti K, Ferreira AM, et al. Striated muscle-specific base editing enables correction of mutations causing dilated cardiomyopathy. Nat Commun. 2023;14(1):3714.

32. Guo M, Xu Y, Dong Z, Zhou Z, Cong N, Gao M, et al. Inactivation of ApoC3 by CRISPR/Cas9 Protects Against Atherosclerosis in Hamsters. Circ Res. 2020;127(11):1456-8.

33. Qiu M, Glass Z, Chen J, Haas M, Jin X, Zhao X, et al. Lipid nanoparticle-mediated codelivery of Cas9 mRNA and single-guide RNA achieves liver-specific in vivo genome editing of Angptl3. Proc Natl Acad Sci U S A. 2021;118(10).

34. Yang P, Lou Y, Geng Z, Guo Z, Wu S, Li Y, et al. Allele-Specific Suppression of Variant MHC With High-Precision RNA Nuclease CRISPR-Cas13d Prevents Hypertrophic Cardiomyopathy. Circulation. 2024;150(4):283-98.

35. Li Q, Su J, Liu Y, Jin X, Zhong X, Mo L, et al. In vivo PCSK9 gene editing using an all-in-one self-cleavage AAV-CRISPR system. Mol Ther Methods Clin Dev. 2021;20:652-9.

36. Cheng Q, Wei T, Farbiak L, Johnson LT, Dilliard SA, Siegwart DJ. Selective organ targeting (SORT) nanoparticles for tissue-specific mRNA delivery and CRISPR–Cas gene editing. Nature Nanotechnology. 2020;15(4):313-20.

37. Ding Q, Strong A, Patel KM, Ng SL, Gosis BS, Regan SN, et al. Permanent alteration of PCSK9 with in vivo CRISPR-Cas9 genome editing. Circ Res. 2014;115(5):488-92.

38. Lee RG, Mazzola AM, Braun MC, Platt C, Vafai SB, Kathiresan S, et al. Efficacy and Safety of an Investigational Single-Course CRISPR Base-Editing Therapy Targeting PCSK9 in Nonhuman Primate and Mouse Models. Circulation. 2023;147(3):242-53.

39. Lu R, Yuan T, Wang Y, Zhang T, Yuan Y, Wu D, et al. Spontaneous severe hypercholesterolemia and atherosclerosis lesions in rabbits with deficiency of low-density lipoprotein receptor (LDLR) on exon 7. EBioMedicine. 2018;36:29-38.

40. Banskota S, Raguram A, Suh S, Du SW, Davis JR, Choi EH, et al. Engineered virus-like particles for efficient in vivo delivery of therapeutic proteins. Cell. 2022;185(2):250-65.e16.

41. Liu S, Li K, Wagner Florencio L, Tang L, Heallen TR, Leach JP, et al. Gene therapy knockdown of Hippo signaling induces cardiomyocyte renewal in pigs after myocardial infarction. Sci Transl Med. 2021;13(600).

42. Ma S, Jiang W, Liu X, Lu WJ, Qi T, Wei J, et al. Efficient Correction of a Hypertrophic Cardiomyopathy Mutation by ABEmax-NG. Circ Res. 2021;129(10):895-908.

43. Wu S, Yang P, Geng Z, Li Y, Guo Z, Lou Y, et al. Base editing effectively prevents early-onset severe cardiomyopathy in Mybpc3 mutant mice. Cell Research. 2024;34(4):327-30.

44. Lebek S, Caravia XM, Chemello F, Tan W, McAnally JR, Chen K, et al. Elimination of CaMKIIδ Autophosphorylation by CRISPR-Cas9 Base Editing Improves Survival and Cardiac Function in Heart Failure in Mice. Circulation. 2023;148(19):1490-504.

45. Lebek S, Chemello F, Caravia XM, Tan W, Li H, Chen K, et al. Ablation of CaMKIIδ oxidation by CRISPR-Cas9 base editing as a therapy for cardiac disease. Science. 2023;379(6628):179-85.

46. Lebek S, Caravia XM, Straub LG, Alzhanov D, Tan W, Li H, et al. CRISPR-Cas9 base editing of pathogenic CaMKIIδ improves cardiac function in a humanized mouse model. J Clin Invest. 2024;134(1).

47. Wang S, Li Y, Xu Y, Ma Q, Lin Z, Schlame M, et al. AAV Gene Therapy Prevents and Reverses Heart Failure in a Murine Knockout Model of Barth Syndrome. Circ Res. 2020;126(8):1024-39.

48. Nishiyama T, Zhang Y, Cui M, Li H, Sanchez-Ortiz E, McAnally JR, et al. Precise genomic editing of pathogenic mutations in RBM20 rescues dilated cardiomyopathy. Sci Transl Med. 2022;14(672):eade1633.

49. Rothgangl T, Dennis MK, Lin PJC, Oka R, Witzigmann D, Villiger L, et al. In vivo adenine base editing of PCSK9 in macaques reduces LDL cholesterol levels. Nat Biotechnol. 2021;39(8):949-57.

50. Wang X, Raghavan A, Chen T, Qiao L, Zhang Y, Ding Q, et al. CRISPR-Cas9 Targeting of PCSK9 in Human Hepatocytes In Vivo-Brief Report. Arterioscler Thromb Vasc Biol. 2016;36(5):783-6.

51. Pan X, Philippen L, Lahiri SK, Lee C, Park SH, Word TA, et al. In Vivo Ryr2 Editing Corrects Catecholaminergic Polymorphic Ventricular Tachycardia. Circ Res. 2018;123(8):953-63.

52. Guo X, Gao M, Wang Y, Lin X, Yang L, Cong N, et al. LDL Receptor Gene-ablated Hamsters: A Rodent Model of Familial Hypercholesterolemia With Dominant Inheritance and Diet-induced Coronary Atherosclerosis. EBioMedicine. 2018;27:214-24.

53. Zha Y, Lu Y, Zhang T, Yan K, Zhuang W, Liang J, et al. CRISPR/Cas9-mediated knockout of APOC3 stabilizes plasma lipids and inhibits atherosclerosis in rabbits. Lipids Health Dis. 2021;20(1):180.

54. Zhang Y, Li H, Min YL, Sanchez-Ortiz E, Huang J, Mireault AA, et al. Enhanced CRISPR-Cas9 correction of Duchenne muscular dystrophy in mice by a self-complementary AAV delivery system. Sci Adv. 2020;6(8):eaay6812.

55. Zuo Y, Zhang C, Zhou Y, Li H, Xiao W, Herzog RW, et al. Liver-specific in vivo base editing of Angptl3 via AAV delivery efficiently lowers blood lipid levels in mice. Cell & Bioscience. 2023;13(1):109.

56. Liu Z, Yang L, Yang Y, Li J, Chen Z, Guo C, et al. ABE-Mediated Cardiac Gene Silencing via Single AAVs Requires DNA Accessibility. Circ Res. 2025;136(3):318-20.

57. Liu Z, Chen S, Xie W, Song Y, Li J, Lai L, et al. Versatile and efficient in vivo genome editing with compact Streptococcus pasteurianus Cas9. Mol Ther. 2022;30(1):256-67.
