# Supplementary material for: Gene editing therapy as a therapeutic approach for cardiovascular diseases in animal models: A scoping review
Source: PLoS One. 2025 Jun 4;20(6):e0325330. doi: 10.1371/journal.pone.0325330 (PMC12136301; doi:10.1371/journal.pone.0325330)
Supplement: Attachment — Submitted filename: Respond_to_Reviewers_comments_auresp_2.docx [file pone.0325330.s005.docx]

**Respond to reviewers’ comments**

I would like to express my gratitude for your thoughtful and constructive feedback on my manuscript. Below, I outline the revisions made to the manuscript based on your insightful remarks.

**Reviewer #2:**

The authors addressed most of my questions properly, except for the exclusion of important short-format papers that are still quite important to the field. Research letters are still original research articles, not simple correspondences.

1. Thank you for highlighting these omissions and providing valuable references. I would like to clarify that some of the suggested articles (e.g., doi: 10.1038/s41422-024-00930-7, 10.1161/CIRCRESAHA.124.325611, and 10.1161/CIRCULATIONAHA.123.065438) were identified during my initial search but were excluded as they are letters or correspondences, which did not meet our inclusion criteria focused on original research.

Re: This is exactly the problem with your exclusion criteria. These excluded research letter papers are still original research and they are peer-reviewed following the same standard as research articles. Their shorter formats simply mean they are focused on single strong points that do not need longer formats. You should still include these papers in your review.

**Respond:**

Thank you for this important clarification. In light of your comment, I have revised inclusion criteria and incorporated the relevant research letters into the final analysis. As a result, the total number of studies included in the review has been updated to 57.
